# Condensin pinches a short negatively supercoiled DNA loop during each round of ATP usage

**DOI:** 10.15252/embj.2022111913

**Published:** 2022-12-19

**Authors:** Belén Martínez‐García, Sílvia Dyson, Joana Segura, Alba Ayats, Erin E Cutts, Pilar Gutierrez‐Escribano, Luís Aragón, Joaquim Roca

**Affiliations:** ^1^ DNA Topology Lab Molecular Biology Institute of Barcelona (IBMB), CSIC Barcelona Spain; ^2^ DNA Motors Group MRC London Institute of Medical Sciences (LMS) London UK

**Keywords:** condensin, DNA supercoil, DNA topology, loop extrusion, SMC complex, Cell Cycle, Chromatin, Transcription & Genomics, Structural Biology

## Abstract

Condensin, an SMC (structural maintenance of chromosomes) protein complex, extrudes DNA loops using an ATP‐dependent mechanism that remains to be elucidated. Here, we show how condensin activity alters the topology of the interacting DNA. High condensin concentrations restrain positive DNA supercoils. However, in experimental conditions of DNA loop extrusion, condensin restrains negative supercoils. Namely, following ATP‐mediated loading onto DNA, each condensin complex constrains a DNA linking number difference (∆Lk) of −0.4. This ∆Lk increases to −0.8 during ATP binding and resets to −0.4 upon ATP hydrolysis. These changes in DNA topology do not involve DNA unwinding, do not spread outside the condensin‐DNA complex and can occur in the absence of the condensin subunit Ycg1. These findings indicate that during ATP binding, a short DNA domain delimited by condensin is pinched into a negatively supercoiled loop. We propose that this loop is the feeding segment of DNA that is subsequently merged to enlarge an extruding loop. Such a “pinch and merge” mechanism implies that two DNA‐binding sites produce the feeding loop, while a third site, plausibly involving Ycg1, might anchor the extruding loop.

## Graphical abstract

Condensin activity produces cyclic alterations in the topology of its interacting DNA, suggesting how this SMC complex generates DNA translocation steps to extrude DNA loops.

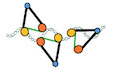

## Introduction

Structural maintenance of chromosomes (SMC) complexes play key roles in the macroscale architecture and dynamics of chromosomes in all domains of life. In bacteria, SMC‐ScpAB and MukBEF promote individualization and segregation of replicated chromosomes (Gruber, 2018; Makela & Sherratt, 2020). In eukaryotes, condensin folds chromatin fibres into rod‐shaped chromatids during mitosis, cohesin mediates sister chromatid cohesion and interphase organisation of chromatin and Smc5/6 is involved in DNA repair (Hirano, 2016; Uhlmann, 2016; Yatskevich *et al*, 2019). Despite these different roles, all SMC complexes have similar ATPase domains and a common architecture, implying they share a core mechanism of action (Hassler *et al*, 2018). In this regard, *in vivo*, *in vitro* and *in silico* studies have converged on the idea that SMC complexes are universal DNA loop extrusion motors (van Ruiten & Rowland, 2018; Datta *et al*, 2020; Davidson & Peters, 2021; Higashi & Uhlmann, 2022). Supporting this notion, biochemical reconstitution assays have proven that condensin and cohesin can extrude DNA loops at high speed (hundreds of bp/s) by consuming little amounts of ATP (Ganji *et al*, 2018; Davidson *et al*, 2019; Kim *et al*, 2019). How SMC complexes dynamically manipulate DNA molecules to extrude DNA loops remains to be elucidated.

The core SMC complex is a large heterotrimeric protein ring formed by two Smc subunits and a kleisin (Haering *et al*, 2002; Gruber *et al*, 2003; Schleiffer *et al*, 2003). In the budding yeast condensin, these are named Smc2, Smc4 and Brn1, respectively (Appendix Fig S1A). Each Smc subunit folds into a 50 nm long antiparallel coiled‐coil that forms a globular “hinge” domain at its apex, whereas the amino and carboxy termini form an ABC‐type ATPase “head” domain at the other end. Smc2 and Smc4 stably dimerize via their hinge domains, while the long and flexible kleisin subunit Brn1 closes the tripartite ring by connecting the two head domains in an asymmetric way. The N‐terminal domain of Brn1 binds to the coiled‐coil “neck” region immediately adjacent to the head of Smc2, while the C‐terminal domain binds to the head tip of Smc4 at a site called “cap”. The condensin complex is completed by two HEAT repeat‐containing proteins Associated With Kleisins (HAWKs), named Ycs4 and Ycg1 in yeast. Ysc4 stably binds to a central region of Brn1 proximal to the neck (HAWK^neck^), whereas Ycg1 binds to a central region proximal to the cap (HAWK^cap^; Uhlmann, 2016; Hassler *et al*, 2018; Yatskevich *et al*, 2019).

Biochemical and structural analyses have exposed a variety of conformational states and DNA interacting modes of SMC complexes (Appendix Fig S1B). The two ATPase heads engage with each other upon binding a pair of ATP molecules between them (Lammens *et al*, 2004). The orientation of the heads in the engaged state spreads apart the coiled‐coil arms (Hassler *et al*, 2019; Vazquez Nunez *et al*, 2021). Upon ATP hydrolysis, the heads disengage and rotate allowing the coiled‐coil SMC arms to align into a rod‐shaped structure (Soh *et al*, 2015; Diebold‐Durand *et al*, 2017). Both in the spread and aligned conformations, the SMC arms can bend at an elbow region, allowing the hinge domain to reach the vicinity of the ATPase heads (Eeftens *et al*, 2016; Burmann *et al*, 2019; Ryu *et al*, 2020). The HAWKs subunits are also highly flexible and dynamic within the complex. In yeast condensin, the Ycs4‐Brn1 module interacts with the two head domains, both in the apo and engaged states; whereas the Ycg1‐Brn1 module is peripheral and more mobile although it can also interact with the other condensin subunits (Hassler *et al*, 2019; Lee *et al*, 2020, 2022). Previous studies identified the hinge as a DNA‐binding module, which presents affinity for single‐ and double‐stranded DNA (Hirano & Hirano, 2006; Griese *et al*, 2010). Another DNA‐binding module is the kleisin‐HAWK^cap^ complex, which secures the DNA with a kleisin belt (Kschonsak *et al*, 2017; Li *et al*, 2018). Lastly, SMC complexes form a central DNA clamping module upon ATP binding, in which DNA is held between the engaged heads and the kleisin‐HAWK^neck^ complex (Higashi *et al*, 2020; Shi *et al*, 2020; Burmann *et al*, 2021; preprint: Shaltiel *et al*, 2021; Lee *et al*, 2022). In addition, DNA can be found topologically or pseudo‐topologically entrapped inside the tripartite ring structure (Ivanov & Nasmyth, 2005; Haering *et al*, 2008; Cuylen *et al*, 2011; Murayama & Uhlmann, 2014) or in other kleisin‐encircled chambers as in the kleisin‐HAWK^cap^ complex (Kschonsak *et al*, 2017; Collier *et al*, 2020; preprint: Shaltiel *et al*, 2021).

Numerous models are currently postulated for the loop extrusion mechanism of SMC complexes. The “walking” and “inchworm” models propose that the coiled‐coils and ATPase heads function like legs that walk or slide along the DNA (Fudenberg *et al*, 2016; Nichols & Corces, 2018). The “pumping” or “segment capture” model speculates that a DNA segment bound at the hinge is pushed towards the head domains via the zipping of the coiled‐coils (Diebold‐Durand *et al*, 2017; Marko *et al*, 2019). The “scrunching” and “swing and clamp” models postulate that motions of SMC arms from the extended to the bent conformations serve to transfer a DNA segment from the hinge to the ATPase heads (Ryu *et al*, 2020; Bauer *et al*, 2021). The “Brownian ratchet” model posits that a DNA clamped via head engagement is only allowed to slip unidirectionally, aided by the motion of the SMC arms (Higashi *et al*, 2021). To this date, it is unknown which, if any, of these models is correct. However, a common trait of these proposed mechanisms is their large impact on the topology of the interacting DNA, which is either pushed, pulled or bent. In this regard, earlier *in vitro* studies had revealed that condensin is able to restrain DNA (+) supercoils in an ATP‐dependent manner (Kimura & Hirano, 1997, 2000; Kimura *et al*, 1999; Takemoto *et al*, 2006; St‐Pierre *et al*, 2009). Since this topological effect required high concentrations and molar ratios of condensin to DNA, its mechanistic significance has not been further investigated. Here, we analysed how condensin alters the topology of the interacting DNA in experimental conditions that sustain DNA loop extrusion (Ganji *et al*, 2018). Surprisingly, we found that during each round of ATP usage, condensin restrains negative DNA supercoils by producing a short left‐handed loop of DNA, which is not in the extruded loop region. We propose a general mechanistic scheme for how SMC complexes generate DNA translocation steps and extrude DNA loops based on these findings.

## Results

### Catalytic amounts of condensin restrain negative DNA supercoils during ATP usage

The linking number (Lk) of double‐stranded DNA in a covalently closed domain equals the sum of the DNA twist (Tw or helical winding of the duplex) and the DNA writhe (Wr or non‐planar bending of the duplex). Accordingly, ∆Lk = ∆Tw + ∆Wr, meaning that any change in Tw and/or Wr constrained by a DNA‐binding factor can be revealed by resetting (relaxing) the Lk of the DNA with a topoisomerase (Appendix Fig S2). Following this notion, several studies had shown that, when relaxed DNA plasmids are incubated with condensin and ATP, topoisomerases increase the Lk of the DNA (Kimura & Hirano, 1997, 2000; Kimura *et al*, 1999; Takemoto *et al*, 2006; St‐Pierre *et al*, 2009). These observations led to the conclusion that condensin restrained DNA (+) supercoils. However, constraining of such (+) supercoils (or more precisely, positive ∆Lk values) required high concentrations (> 50 nM) and molar ratios of condensin to DNA (> 1 complex/100 bp). Hence, we asked whether low concentrations and molar ratios of condensin, as those supporting DNA loop extrusion, could also produce measurable ∆Tw and ∆Wr deformations in the DNA. To this end, we incubated different amounts of the purified budding yeast condensin (Appendix Fig S3) with a relaxed DNA plasmid (4.3 kb) in presence of vaccinia virus topoisomerase I (Topo I). To determine ∆Lk changes accurately, we examined the resulting distribution ladders of Lk topoisomers in 1D or 2D agarose gel electrophoreses containing calculated amounts of chloroquine (Appendix Fig S4).

**Figure 1:**
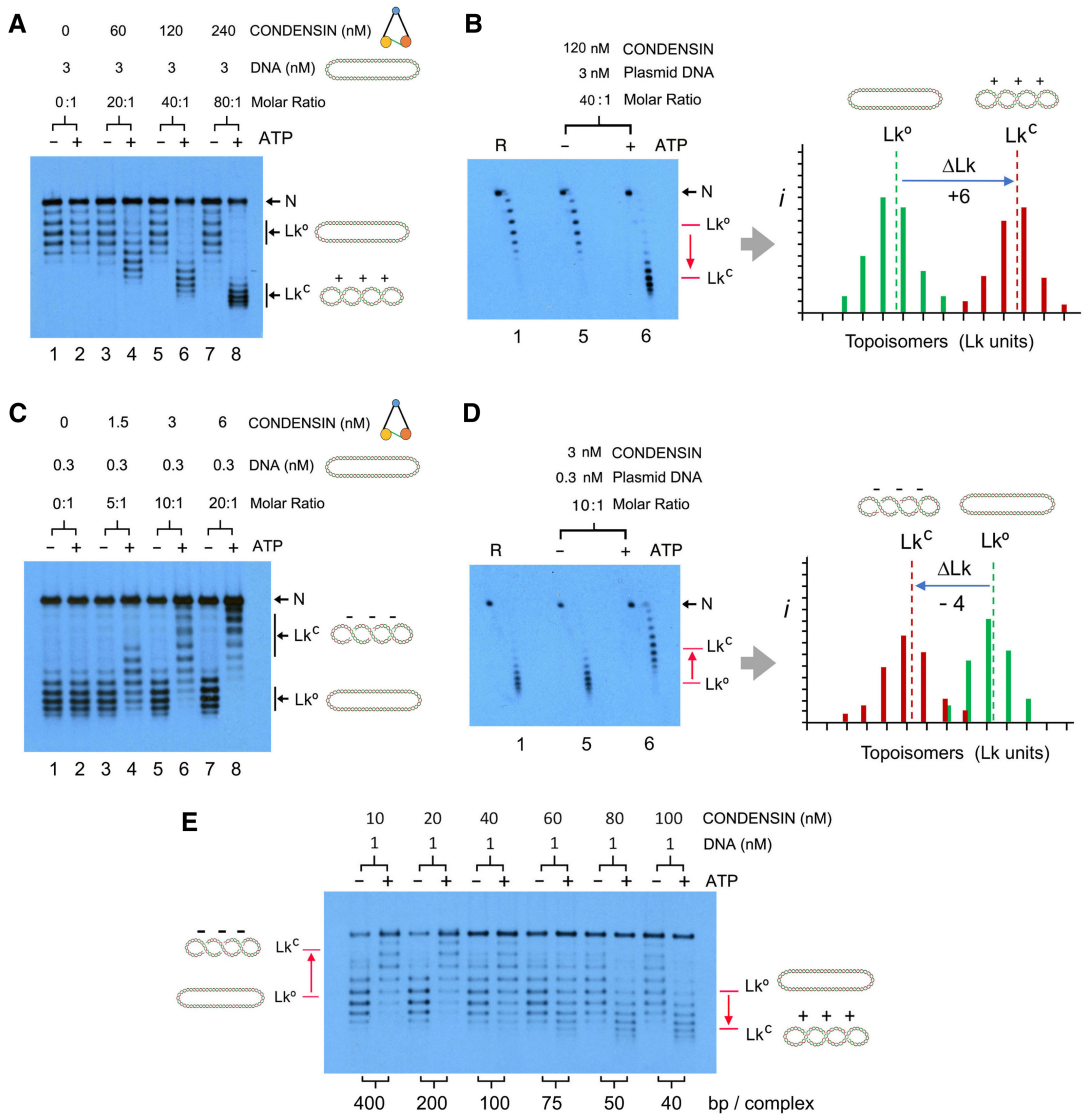
Effect of condensin concentration to restrain DNA supercoils Relaxed DNA (3 nM), condensin (0, 60, 120, 240 nM) and Topo I (1 unit) were mixed and incubated with or without ATP (1 mM) at 30°C for 30 min. DNA electrophoresis contained 0.1 μg/ml chloroquine. In all gels, N denotes nicked circles; Lk°, the input Lk distribution of relaxed DNA; and Lk^C^, the Lk distribution restrained by condensin.2D‐gel of the preceding samples in lane 1 (relaxed DNA, R) and lanes 5 and 6 (condensin 120 nM ± ATP). Electrophoresis contained 0.1 and 1 μg/ml chloroquine in the first and second dimension, respectively. The histogram shows the relative intensity (*i*) of individual topoisomers of the Lk distributions resolved in lane 1 (green) and lane 6 (red). Lk° and Lk^C^ denote the midpoint of each *Lk* distribution; and ∆Lk, the difference (Lk units) between them.Experiment conducted as in (A), but reducing the concentration of DNA (0.3 nM) and condensin (0, 1.5, 3, 6 nM). DNA electrophoresis contained 0.4 μg/ml chloroquine.2D‐gel of the preceding sample in lane 1 (relaxed DNA, R), and lanes 5 and 6 (condensin 3 nM ± ATP). Electrophoresis contained 0.4 and 1 μg/ml chloroquine in the first and second dimension, respectively. The histogram shows relative Lk intensities (*i*) of lanes 1 and 6, indicating Lk°, Lk^C^ and ∆Lk.Experiment conducted as in (A), but mixing DNA (1 nM) with intermediate condensin concentrations (10–100 nM). DNA electrophoresis contained 0.2 μg/ml chloroquine. The length of DNA (bp) available per condensin complex in each reaction is indicated.

**Figure EV1:**
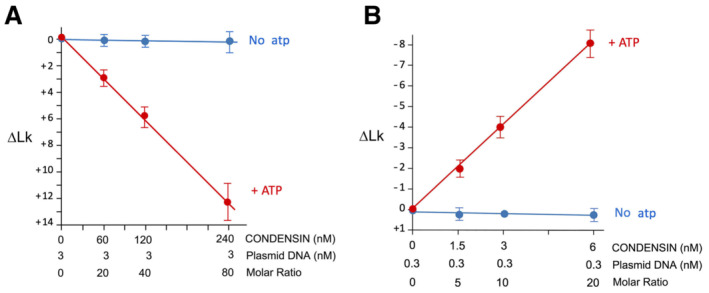
∆Lk values restrained at high and low condensin concentrations (relative to Fig 1) Plot of positive ∆Lk values restrained by high concentrations and molar ratios of DNA and condensin with and without ATP (mean ± SD, three technical replicates conducted as in Fig 1A).Plot of negative ∆Lk values restrained by low concentrations and molar ratios of DNA and condensin with and without ATP (mean ± SD from three independent experiments conducted as in Fig 1C).

First, we tested high concentrations and molar ratios of condensin to DNA (Fig 1A). In the absence of ATP, Topo I did not significantly alter the Lk of the relaxed DNA (R), even when mixed with high concentrations (240 nM) and molar ratios (80:1) of condensin. However, upon addition of ATP, Topo I increased the Lk of the plasmid proportionally to the amount of condensin (Figs 1A and EV1), in agreement with the restraining of (+) supercoils observed in earlier studies (Kimura & Hirano, 1997, 2000; Kimura *et al*, 1999; Takemoto *et al*, 2006; St‐Pierre *et al*, 2009). We determined that the ∆Lk restrained per condensin was about +0.15 (+6/40) (Fig 1B), which denoted that each holo‐complex might be stabilising a slight overtwisting (∆Tw ≈ +0.15) or right‐handed bending (∆Wr ≈ +0.15) of the DNA (Appendix Fig S5; Vologodskii & Cozzarelli, 1994; Segura *et al*, 2018).

**Figure EV2:**
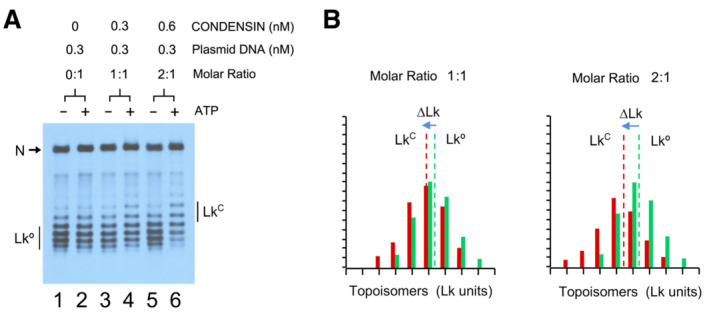
Restraining of DNA supercoils with low molar ratios of condensin (relative to Fig 1) Relaxed DNA (0.3 nM), condensin (0, 0.3, 0.6 nM) and Topo I (1 unit) were mixed in 25 mM Tris–HCl pH 7.5, 25 mM NaCl, 5 mM MgCl_2_, 1 mM DTT, with/without ATP (1 mM). Incubations proceeded at 30°C for 30 min. DNA electrophoresis was at 2.5 V/cm for 20 h in 0.7% agarose and TBE buffer containing 0.4 μg/ml chloroquine. N, nicked circles. Lk°, input distribution of Lk topoisomers of relaxed DNA. Lk^C^, resulting distribution of Lk topoisomers.The histograms compare the relative intensity of individual topoisomers of the Lk distributions in lanes 3 and 4 (condensin:DNA molar ratio 1:1); and lanes 5 and 6 (molar ratio 2:1). Lk° and Lk^C^ denote the midpoint of each Lk distribution and ∆Lk the difference between them.

Next, we tested reducing the concentration and molar ratios of condensin to DNA over 10‐fold, thus mimicking the reaction settings that support DNA loop extrusion (Ganji *et al*, 2018; Kim *et al*, 2019). In these conditions, condensin activity did no longer restrain (+) supercoils. Instead, it restrained (−) supercoils (Figs 1C and EV1). Namely, Topo I reduced the Lk of the DNA, producing a ∆Lk of about −0.4 (−4/10) per condensin complex in an ATP‐dependent manner (Fig 1D). This topological effect was observable with molar ratios as little as one condensin complex per plasmid (Fig EV2) and thus denoted a significant untwisting (∆Tw ≈ −0.4) or left‐handed bending (∆Wr ≈ −0.4) of the interacting DNA (Appendix Fig S5). To observe the transition from restraining (+) to (−) supercoils, we tested intermediate molar ratios of condensin to DNA (Fig 1E). Apparently, such transition occurred when the DNA length available per condensin complex was about 100 bp. Shorter lengths led to the restrain of (+) supercoils, whereas larger lengths allowed the restrain of (−) ones. As in the case of DNA loop extrusion, restraining of (−) supercoils was optimal in low or moderate salt buffers (25–100 mM NaCl/KCl) containing divalent cations (1–5 mM MgCl_2_; Appendix Fig S6). Restraining of (−) supercoils was robust at a physiological pH (7.5) and at several temperatures (15–45°C; Appendix Fig S7).

### Restrained negative supercoils persist after ATP hydrolysis and increase during ATP binding

**Figure 2:**
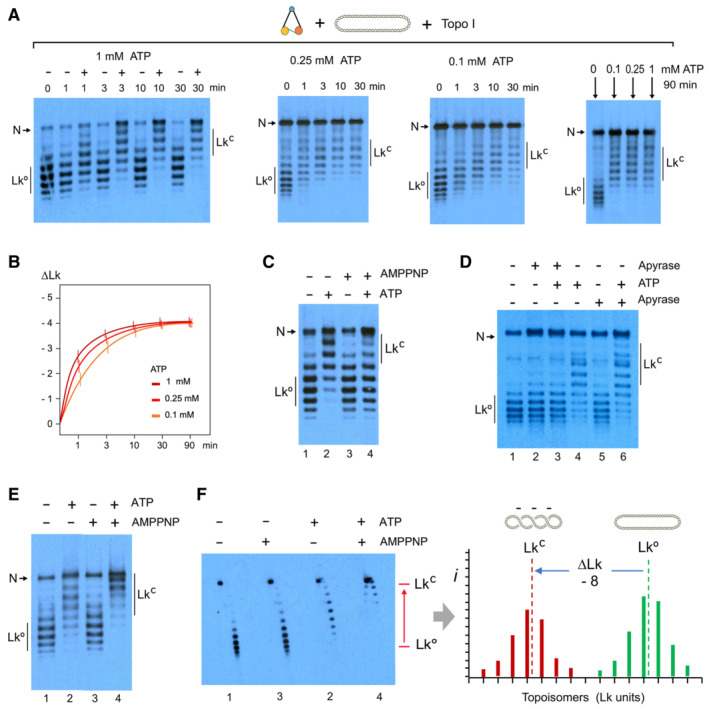
Role of ATP in condensin restraining of negative DNA supercoils Relaxed DNA (0.3 nM), condensin (3 nM) and Topo I (1 unit) were incubated at 30°C for indicated time periods (0–90 min) in the presence of different concentrations of ATP (1, 0.25, 0.1 mM).Plot of ∆Lk values (mean ± SD, three technical replicates) constrained in the preceding experiment.DNA, condensin and Topo I (mixed as in A) were incubated at 30°C without nucleotides for 10 min (lane 1), with ATP 1 mM for 10 min (lane 2), AMPPNP 2 mM for 10 min (lane 3) and AMPPNP 2 mM for 10 min followed by ATP 1 mM for 10 min (lane 4).DNA, condensin and Topo I (mixed as in A) were incubated at 30°C without nucleotides for 10 min (lane 1), with Apyrase for 10 min (lane 2), Apyrase and ATP 1 mM for 10 min (lane 3), ATP 1 mM for 10 min (lane 4), no nucleotide for 10 min followed by Apyrase for 60 min (lane 5), ATP 1 mM for 10 min followed by Apyrase for 60 min (lane 6).DNA, condensin and Topo I (mixed as in A) were incubated at 30°C without nucleotides for 20 min (lane 1), with ATP 1 mM for 20 min (lane 2), AMPPNP 2 mM for 20 min (lane 3), ATP 1 mM for 10 min followed by AMPPNP 2 mM for 10 min (lane 4).2D‐gel of the samples in (E) (lanes 1 to 4) and histogram of Lk intensities (*i*) of lanes 1 and 4, indicating Lk°, Lk^C^ and ∆Lk. Data information: DNA electrophoreses were conducted and labelled as in Fig 1.

Condensin restraining of DNA (−) supercoils occurred quickly (1–10 min) following ATP addition and reached a plateau (∆Lk ≈ −0.4) irrespective of the initial concentration of ATP (0.1–1 mM; Fig 2A and B). Conversely, incubation of condensin and DNA in the presence of AMPPNP, a non‐hydrolysable ATP analogue, barely altered the topology of DNA. Yet, preincubation of condensin and DNA with AMPPNP precluded the effect of ATP subsequently added to the reactions (Fig 2C). Therefore, restraining of (−) supercoils required the hydrolysis of the bound ATP.

To test whether restraining of (−) supercoils relied on continuous cycles of ATP usage, we incubated condensin and DNA in the presence of ATP and, afterwards, we added Apyrase or Alkaline Phosphatase to exhaust the ATP. Both ATP hydrolases produced similar results (Fig 2D and Appendix Fig S8). As expected, condensin did not restrain (−) supercoils when the ATP hydrolases were added at the beginning of the incubations. However, when the hydrolases were added after 10 min of ATP usage, the (−) supercoils constrained by condensin persisted during extended time periods (60 min). Therefore, continuous use of ATP was not necessary to maintain DNA (−) supercoils restrained.

To further assess whether the restraining of (−) supercoils does not require continuous cycles of ATP hydrolysis, we incubated condensin and DNA with ATP for 10 min and then we added AMPPNP to quench ATP usage. Surprisingly, such addition of AMPPNP increased by twofold the amount of (−) supercoils restrained by condensin (Fig 2E). Namely, each condensin complex restrained a ∆Lk of about −0.8 (−8/10) (Fig 2F). Such ∆Lk of −0.8 could denote the untwisting of nearly one helical turn of DNA (∆Tw ≈ −0.8) or the stabilisation of a compact left‐handed coil of DNA (∆Wr ≈ −0.8) (Appendix Fig S5). This large ∆Lk restraint contrasted with the minimal effect of AMPPNP in the absence of ATP. Therefore, only following the initial cycles of ATP hydrolysis, nucleotide binding produces a conformation that further enhances the restraining of (−) supercoils.

### Condensin does not unwind DNA to restrain negative supercoils

**Figure 3:**
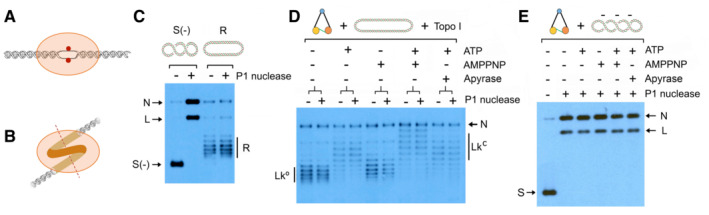
Endonuclease assays to test DNA unwinding by condensin DNA unwinding (∆Tw < 0) restrains negative ∆Lk.Left‐handed bending or looping of DNA (∆Wr < 0) restrains negative ∆Lk.Negatively supercoiled (0.3 nM) and relaxed DNA (0.3 nM) were incubated with/without nuclease P1 for 10 min at 30°C in condensin reaction buffer.Relaxed DNA (0.3 nM), condensin (3 nM) and Topo I were mixed with/without nuclease P1 and incubated at 30°C with no nucleotide for 30 min, ATP 1 mM for 30 min, AMPPNP 2 mM for 30 min, ATP for 20 min followed by AMPPNP for 10 min or ATP for 20 min followed by Apyrase for 10 min.Experiment conducted as in (D), but using (−) supercoiled DNA and without Topo I. Following the incubation with nucleotides for 30 min, nuclease P1 was added for 5 min. Data information: Supercoiled (S), relaxed (R), nicked (N) and linear DNA signals are indicated.

Condensin could restrain negative ∆Lk values either by unwinding the DNA duplex (∆Tw < 0) or producing a left‐handed DNA turn or loop (∆Wr < 0) or combining both types of deformations (Fig 3A and B). To test whether condensin was unwinding the DNA during ATP usage, we attacked with single‐stranded DNA endonucleases the condensin‐DNA complexes that restrained (−) supercoils. We chose Nuclease P1 because this enzyme does not produce any nick in relaxed DNA, whereas it is very proficient in nicking negatively supercoiled (i.e., untwisted) DNA (Fig 3C and Appendix Fig S9). Thus, we mixed condensin, relaxed DNA plasmid, Topo I and Nuclease P1. Then, we supplemented the mixtures with either ATP, AMPPNP, ATP followed by AMPPNP or ATP followed by Apyrase. Following 30 min incubations, Nuclease P1 did not nick at all the DNA plasmids in which condensin was restraining (−) supercoils (Fig 3D). We discarded that condensin could be inhibiting the nuclease by conducting a similar experiment, in which the input DNA was negatively supercoiled and Topo I was not included in the reactions. In this experiment, the nuclease was added at the end of the incubations and rapidly nicked all the plasmids (Fig 3E). Therefore, unless condensin was protecting an unwound region of DNA from the nuclease attack, the ∆Lk values (−0.4 and −0.8) restrained by condensin were likely reflecting the configuration of a left‐handed DNA turn or loop (Appendix Fig S5).

### Restraining of supercoils correlates with the stability of condensin‐DNA complexes

**Figure 4:**
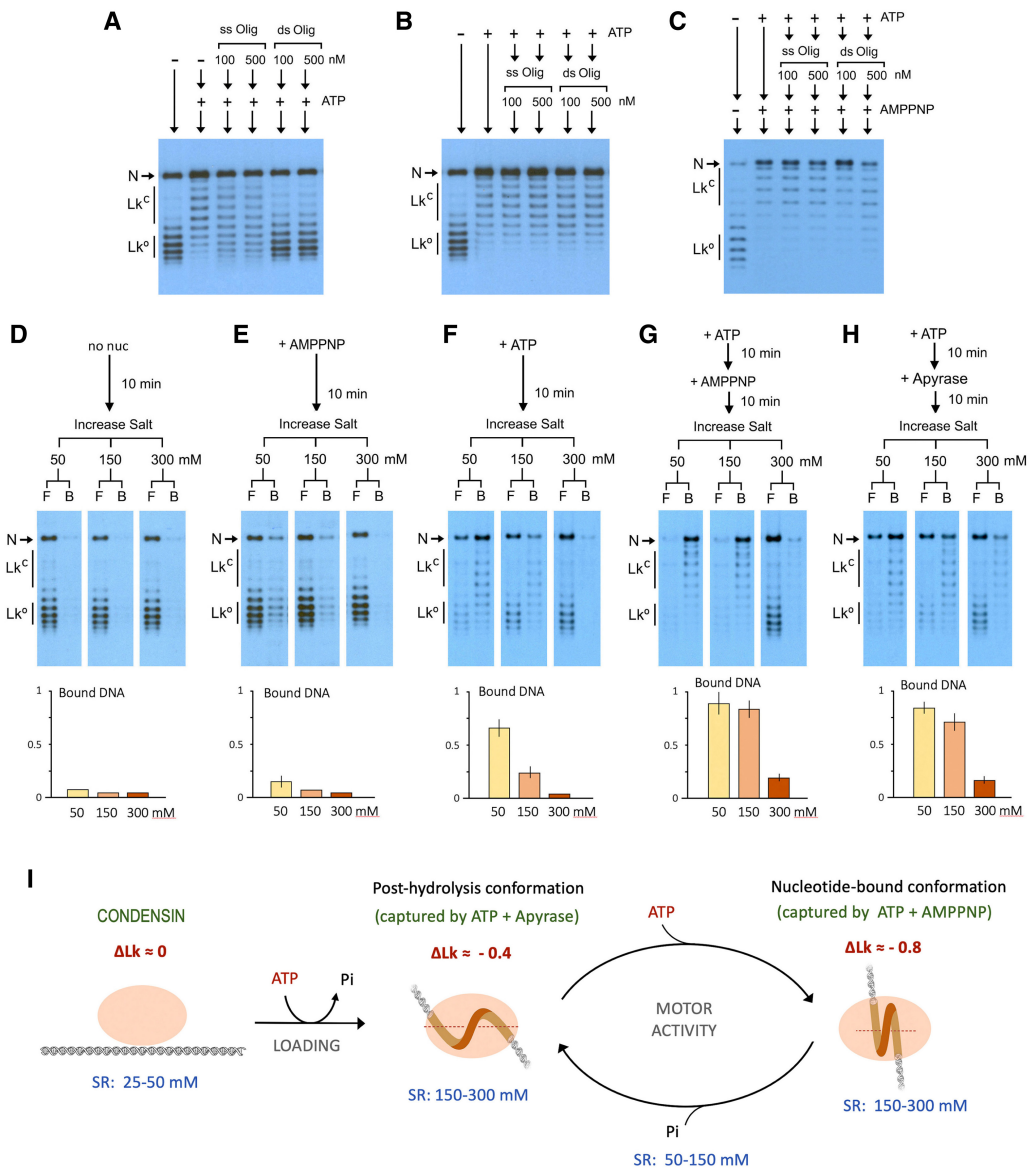
Stability of condensin‐DNA complexes during the restraining of DNA supercoils Relaxed DNA (0.3 nM), condensin (3 nM) and Topo I (1 unit) were mixed with ss‐oligos or ds‐oligos (100 and 500 nM). Following incubation for 10 min at 30°C, ATP (1 mM) was added and incubations continued for 10 min.Experiment as in (A), but first adding ATP for 10 min and afterwards the oligonucleotides for 10 min.Experiment as in (B), but after the 10 min incubation with oligonucleotides, AMPPNP (2 mM) was added and incubations continued for 10 min.Relaxed DNA (0.3 nM), condensin (3 nM) and Topo I (1 unit) were incubated at 30°C for 10 min. Reactions were then split into thirds to which NaCl concentration was raised to 50, 150 and 300 mM. Salt‐resistant condensin‐DNA complexes were immobilised to His‐Tag magnetic beads and the fractions of free (F) and bound (B) DNA recovered. The plot shows the fractions of bound DNA (mean ± SD, three technical replicates).As in (D), but containing AMPPNP (2 mM).As in (D), but containing ATP (1 mM).As in (D), but containing ATP (1 mM) for 10 min followed by AMPPNP (2 mM) for 10 min.As in (D) but containing ATP (1 mM) for 10 min followed by Apyrase for 10 min.Condensin‐DNA conformations inferred from the restrained ∆Lk values and the salt resistance (SR) of complexes during distinct stages of ATP usage.

Since condensin‐DNA interactions must be dynamic, we examined the stability of DNA (−) supercoils restrained by condensin. To this end, we tested the interaction of condensin with plasmid DNA in the presence of a molar excess of single‐ or double‐stranded DNA oligonucleotides (ss‐ or ds‐oligos). First, we incubated condensin (3 nM), plasmid (0.3 nM) and Topo I in low salt buffer (25 mM). Afterwards, we added competitor DNAs (100 or 500 nM) and, lastly, ATP. Ds‐oligos completely abolished the capacity of condensin to restrain (−) supercoils, whereas ss‐oligos produced a lesser effect (Fig 4A). Importantly, oligonucleotides did not affect Topo I activity (Appendix Fig S10). Therefore, prior to ATP usage, condensin‐DNA interactions must be weak since they were overtaken by competitor DNAs. However, when we incubated condensin, plasmid DNA and Topo I in presence of ATP for 10 min and then added the competitor DNAs, neither ds‐ nor ss‐oligos affected the capacity of condensin to maintain (−) supercoils constrained (Fig 4B). Therefore, condensin‐DNA interactions restraining (−) supercoils are either enduring or are reinstated very quickly during each round of ATP usage. We also found that the presence of competitor DNAs did not preclude the extra ∆Lk restraint produced when ATP usage was quenched by AMPPNP (Fig 4C). Only high concentrations of ds‐oligos (500 mM) slightly reduced the extra ∆Lk restraint. Therefore, the transition from restraining ∆Lk −0.4 to −0.8 was most likely due to a conformational change of the condensin‐DNA complex rather than to the occupancy of an additional DNA‐binding site.

To further assess the stability of condensin‐DNA interactions, we examined the ability of condensin to maintain (−) supercoils restrained under different ionic strength conditions. We incubated condensin, plasmid and Topo I in a low salt buffer (NaCl 25 mM) in the absence or presence of nucleotides. Following the incubations, we increased the salt concentration to 50, 150 or 300 mM and then immobilised condensin to magnetic beads such that we could recover the fractions of free (F) and condensin‐bound DNA (B). In the absence of ATP (Fig 4D) or presence of AMPPNP (Fig 4E), nearly all plasmid molecules were free in 50 mM salt. In contrast, when ATP was present in the reactions (Fig 4F), most plasmids were found in the condensin‐bound fraction. However, the fraction of bound plasmids was markedly reduced when the ionic strength was raised to 150 mM NaCl. Next, we examined the reactions in the presence of ATP but subsequently quenched by the addition of AMPPNP (Fig 4G). In this case, most plasmids remained bound to condensin when the salt concentration was increased to 150 mM. Only when the salt was raised to 300 mM, the majority of plasmids appeared in the unbound fraction. Lastly, we examined the reactions initiated in the presence of ATP but subsequently exhausted by the addition of Apyrase (Fig 4H). Here again, condensin‐DNA complexes resisted 150 mM salt; and only after raising the salt to 300 mM, most plasmids were found to dissociate. Importantly, in all cases, the DNA plasmids that remained bound to condensin presented restrained (−) supercoils. Conversely, when DNA plasmids were dissociated from condensin, their (−) supercoils become unconstrained and relaxed by Topo I. Therefore, the overall changes in complex stability and capacity to restrain supercoils denoted distinct conformational stages of the condensin‐DNA complex during ATP usage (Fig 4I).

### Condensin‐mediated changes in DNA topology do not spread outside the condensin‐DNA complex

**Figure 5:**
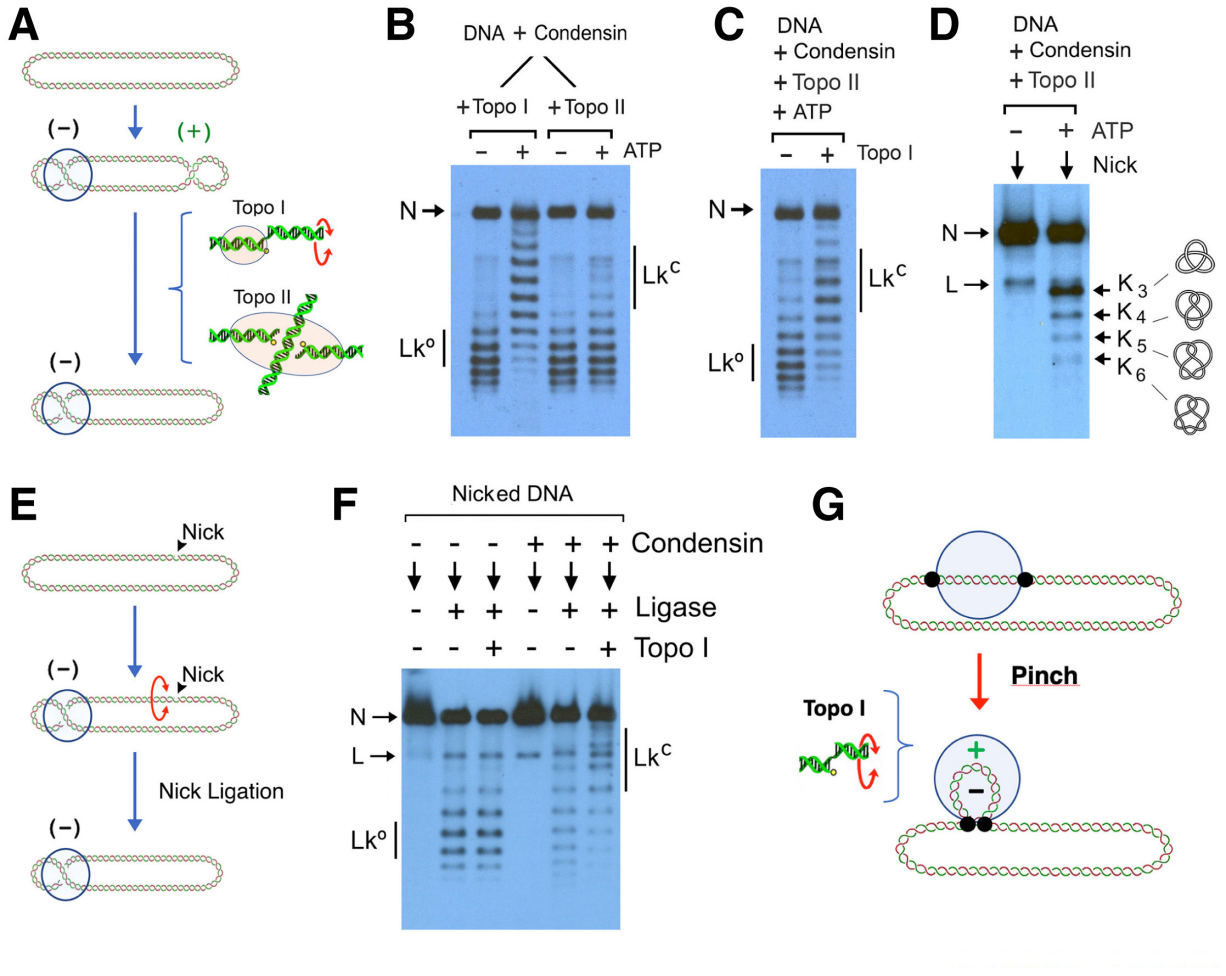
DNA supercoiling does not spread outside the condensin‐DNA complex Either the DNA‐strand rotation mechanism of Topo I or the DNA‐cross inversion mechanism of Topo II could relax compensatory (+) supercoils outside the condensin‐DNA complex.Relaxed DNA (0.3 nM) and condensin (3 nM) were mixed with Topo I or Topo II (1 unit). Upon addition of ATP (1 mM), incubations proceeded at 30°C for 30 min.DNA, condensin, and Topo II were mixed as in (B). Upon addition of ATP (1 mM), incubations proceeded at 30°C for 10 min. Topo I was added to one half of the mixtures and incubations continued for 10 min.DNA, condensin and Topo II were mixed as in (B). Following incubation at 30°C for 10 min with or without ATP, DNA was recovered and nicked to reveal the occurrence of DNA knots. K_3_ to K_6_ denote knots with 3–6 irreducible DNA crossings.The presence of a nick would relax the compensatory (+) supercoils outside the condensin‐DNA complex; and subsequent nick ligation would capture the restrained (−) supercoils.Nicked DNA (0.3 nM) was mixed without or with condensin (3 nM) in the presence of ATP (1 mM). Following incubations at 30°C for 20 min, mixtures were supplemented with T4 DNA Ligase and Topo I (as indicated) and incubation continued for 10 min.If two DNA‐binding sites delimit a short topological domain, which is subsequently pinched into a (−) supercoil, the compensatory (+) supercoiling would remain within such topological domain.

Our experiments were done using Topo I, which is a type‐1B topoisomerase that transiently cleaves one strand of duplex DNA and allows free rotation of the other strand in either direction to release (+) or (−) DNA helical tension (Champoux, 2001). Accordingly, when condensin restrained (−) supercoils, Topo I relaxed the compensatory (+) supercoiling and thus reduced the Lk of DNA (Fig 5A). Then, we expected that any topoisomerase able to relax (+) supercoiling should also reduce the Lk of DNA. To this end, we tested topoisomerase II of budding yeast (Topo II), which is a type‐2A topoisomerase that passes one segment of duplex DNA through a transient double‐strand break produced in another segment in an ATP‐dependent manner (Fig 5A; Champoux, 2001). Surprisingly, in contrast to Topo I, Topo II did not produce any significant change in the Lk of the DNA during condensin activity (Fig 5B). We discarded that Topo II could be abrogating the activity of condensin because the Lk of the plasmid was reduced when Topo I was added to the mixtures already containing Topo II (Fig 5C). We also excluded that condensin could be inhibiting Topo II activity since Topo II was able to relax supercoiled plasmids subsequently added to the condensin‐DNA mixtures (Appendix Fig S11). Moreover, rather than relaxing the DNA during condensin activity, Topo II produced DNA knots (K_3_, K_4_, K_5…_; Fig 5D), which likely reflected the entanglement of intramolecular DNA loops extruded by condensin.

**Figure EV3:**
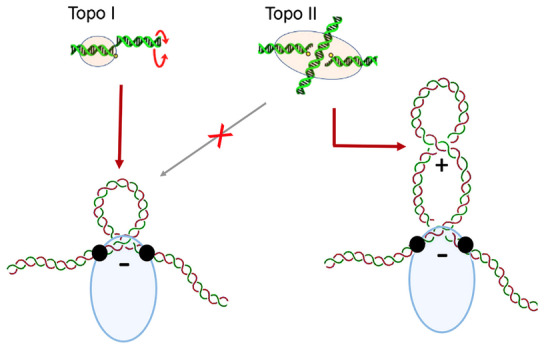
Capacity of Topo I and Topo II to relax short DNA domains (relative to Fig 5) The strand‐rotation mechanism of Topo I requires interacting only with a short segment of DNA (< 30 bp) to relax DNA helical tension (Champoux, 2001). Conversely, DNA relaxation by the cross‐inversion mechanism of Topo II requires a DNA domain long enough to facilitate the juxtaposition of two intramolecular DNA segments. Then, in the case of the DNA loop restricted by condensin, the loop length should accommodate both the restrained (−) supercoil and the compensatory (+) supercoil to be relaxed by Topo II.

The incapacity of Topo II to reduce the Lk of the DNA indicated that, when condensin restrains (−) DNA supercoils, the compensatory (+) supercoiling does not spread outside the condensin‐DNA complex. Hence, Topo II cannot find DNA crossovers to relax the DNA. To further explore this limitation, we conducted an experiment in which condensin restrained (−) supercoils in singly‐nicked DNA plasmids (Fig 5E). If compensatory (+) supercoiling were dissipated by the nick, subsequent nick ligation would capture the ∆Lk restrained by condensin. We found that nick ligation did not result in a reduction of Lk comparable to that produced by Topo I in covalently closed DNA (Fig 5F). This observation corroborated that the compensatory (+) supercoiling does not spread outside the condensin‐DNA complex. Therefore, the restrained (−) supercoils and the compensatory (+) supercoiling must occur within a DNA topological domain delimited by condensin (Fig 5G). This topological domain, which is pinched by condensin into a left‐handed loop, must be large enough for the Topo I mechanism to relax the DNA, but not large enough to form and expose a compensatory (+) DNA crossover that could be relaxed by Topo II (Fig EV3). The low probability of having a single nick allocated within this short domain explained why nick ligation did not capture negative ∆Lk values to the same extent as Topo I.

### Condensin activity does not generate twin supercoiled loops

**Figure 6:**
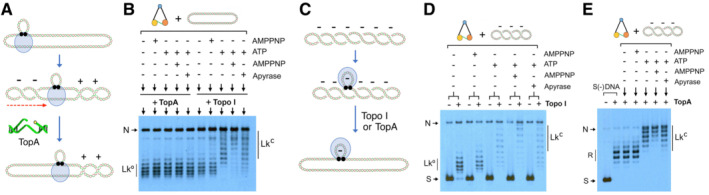
Condensin does not generate twin supercoiled loops while restraining negative supercoils If condensin translocation produces twin (+) and (−) supercoiled domains, (+) supercoils would accumulate upon relaxation of (−) supercoils with TopA.Relaxed DNA (0.3 nM) was mixed with condensin (3 nM). Following incubation in absence or presence of the indicated nucleotides (ATP 1 mM for 30 min, AMPPNP 2 mM for 30 min, ATP for 20 min followed by AMPPNP for 10 min or ATP for 20 min followed by Apyrase for 10 min), TopA was added to one half of each reaction and Topo I to the other half. Incubations continued for 10 min.Relaxation of unconstrained (−) supercoils by either Topo I or TopA could reveal condensin ATP‐dependent stabilisation of pre‐existing (−) supercoils.Experiment conducted as in B but starting with (−) supercoiled DNA instead of relaxed DNA. Following incubation in presence of the indicated nucleotides (as in B) Topo I was added to one half of each mixture and incubations continued for 10 min.Experiment conducted as in D but adding TopA instead of Topo I.

While the above results indicated that compensatory (+) supercoiling does not diffuse outside the condensin complex, they did not exclude that condensin could be generating twin supercoiled loops. Namely, in addition to restraining (−) supercoils, condensin translocation could generate (+) supercoiling of DNA in front of the moving complex and (−) supercoiling behind it (Fig 6A). As the amount of (+) and (−) supercoils in such twin domains would be equal, only an asymmetric relaxation would produce a change in the DNA Lk. To test this possibility, we relaxed the DNA with *Escherichia coli* topoisomerase I (TopA), a type‐1A topoisomerase that relaxes (−) but not (+) DNA supercoils (Champoux, 2001). Accordingly, the relaxation of the (−) supercoiled domain would increase the DNA's Lk. We found that TopA did not increase the Lk of the relaxed plasmids incubated with condensin and ATP (Fig 6B), indicating the absence of twin supercoiled loops.

**Figure EV4:**
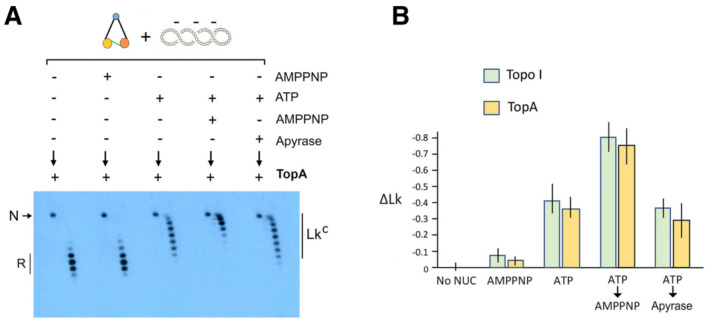
Relaxation of unconstrained (−) supercoils with TopA (relative to Fig 6) (−) Supercoiled DNA (0.3 nM) was mixed with condensin (3 nM) in 25 mM Tris–HCl pH 7.5, 25 mM NaCl, 5 mM MgCl_2_, 1 mM DTT. Following incubation in the absence or presence of the indicated nucleotides (ATP 1 mM for 30 min, AMPPNP 2 mM for 30 min, ATP for 20 min followed by AMPPNP for 10 min or ATP for 20 min followed by Apyrase for 10 min), TopA was added to relax unconstrained (−) supercoils. Incubations continued for additional 10 min. 2D‐gel electrophoresis was done as in Fig 1D.Comparison of ∆Lk values (mean ± SD, three technical replicates) restrained by condensin when unconstrained (−) supercoils were relaxed by Topo I or TopA.

The activity of TopA, however, corroborated the capacity of condensin to restrain (−) supercoils in another experimental setting. Namely, we incubated condensin with (−) supercoiled DNA instead of relaxed DNA and, only at the end of the reactions, we added topoisomerases to relax the unrestrained (−) supercoils (Fig 6C). As expected, in these conditions, Topo I produced ∆Lk values similar to those produced when the input DNA was already relaxed (Fig 6D). Remarkably, when we used TopA instead of Topo I to relax the unrestrained (−) supercoils, the ∆Lk results were also similar to those obtained with Topo I during the different stages of ATP usage (Figs 6E and EV4). Importantly, these results also indicated that DNA supercoiling energy cannot replace the need for ATP to restrain (−) supercoils.

### Ycg1 subunit is not required for condensin to restrain negative supercoils

**Figure 7:**
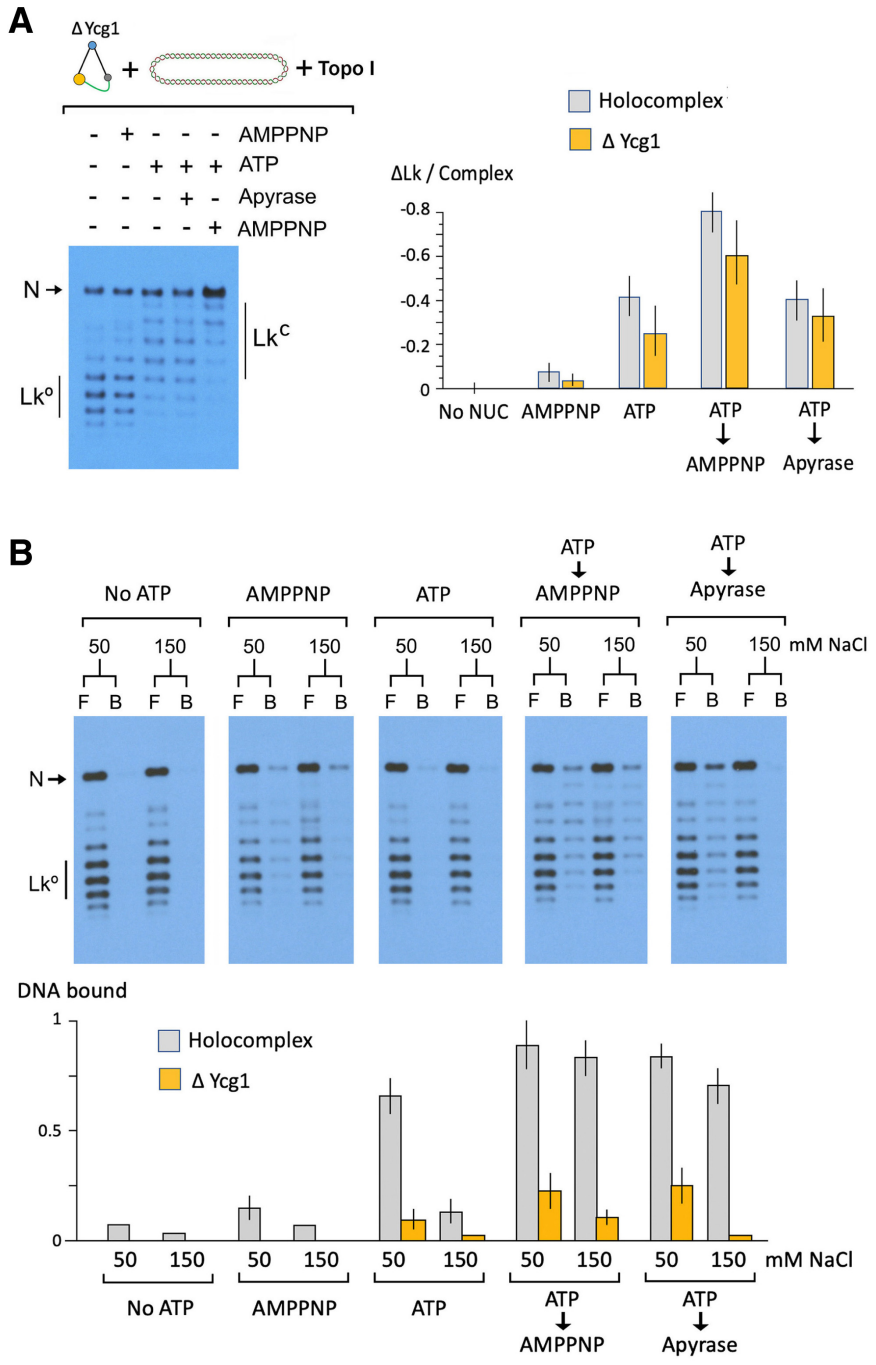
Ycg1 subunit is not required to restrain negative supercoils Relaxed DNA (0.3 nM), ∆Ycg1 condensin (3 nM) and Topo I were incubated at 30°C with no nucleotide for 30 min, ATP 1 mM for 30 min, AMPPNP 2 mM for 30 min, ATP for 20 min followed by AMPPNP for 10 min or ATP for 20 min followed by Apyrase for 10 min. The plot (mean ± SD, three technical replicates) compares the ∆Lk values restrained by the condensin holo‐complex and the ∆Ycg1 condensin.Salt resistance of the ∆Ycg1 condensin‐DNA complex. Experiments were conducted as in Fig 4D–H. The plot (mean ± SD, three technical replicates) compares the fractions of DNA bound to the holo‐complex and to the ∆Ycg1 tetramer.

The capacity of condensin to pinch a small topological domain of DNA into a left‐handed loop implies the concerted action of two separate DNA‐binding sites within the complex. Then, we tested if the absence of the Ycg1 subunit, one of the main DNA‐binding modules of condensin, would impair the capacity to restrain (−) supercoils. Surprisingly, the tetrameric (∆Ycg1) complex (Appendix Fig S3) was able to restrain (−) supercoils following a similar trend to that of the holo‐complex (Fig 7A). Stabilisation of negative ∆Lk values occurred upon ATP hydrolysis, it was enhanced during AMPPNP binding and persisted upon ATP exhaustion with Apyrase. However, the ∆Ycg1 complex exhibited little salt resistance in comparison with the holo‐complex (Fig 7B). Whereas the condensin‐DNA holo‐complex was able to resist salt concentrations over 150 mM during the ATP‐bound stage (ATP followed by AMPNP) and during the post‐hydrolysis stage (ATP followed by Apyrase), the ∆Ycg1 complex could barely hold the DNA even at low salt concentrations (50 mM). These results indicated that Ycg1 plays a role in keeping condensin attached to DNA but not in the restraining of (−) supercoils, which would involve weaker or transient DNA interactions elsewhere in the complex.

## Discussion

**Figure EV5:**
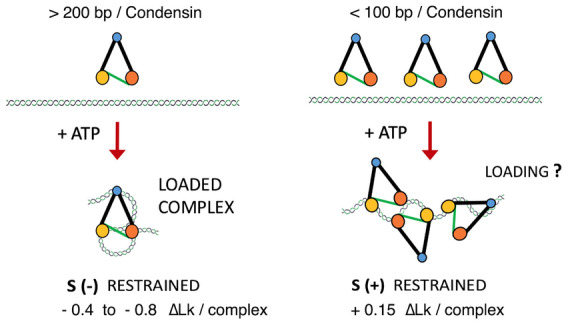
Effect of the accessible DNA length on complex loading and restraining of supercoils (relative to Fig 1) ATP‐mediated loading of condensin leads to the confinement of 100–200 bp of DNA per complex (Bazett‐Jones *et al*, 2002). Accordingly, low molar ratios of condensin to DNA (> 200 bp/complex) would allow complete loading events, which produce the restraining of (−) supercoils. High molar ratios of condensin (< 100 bp/complex) would impair proper loading and produce other DNA deformations that restrain (+) supercoils.

Before the DNA loop extrusion activity of SMC complexes came to light, the capacity of condensin to restrain DNA (+) supercoils was postulated as a mechanism that compacted mitotic chromosomes (Hirano, 2014). However, here we showed that such restraint of (+) supercoils only occurs by using high molar ratios of condensin to DNA (> 1 condensin/100 bp; Fig 1A and E). When DNA is mixed with such an excess of condensin, far from physiological ratios of about one condensin per 10 Kb (Wang *et al*, 2005), the extrusion of DNA loops is impracticable (Kim *et al*, 2020). By reducing the amount of condensin to one or few complexes per DNA plasmid, as in DNA loop extrusion assays, we found that condensin activity restrains (−) supercoils (Fig 1C and E). Since ATP‐mediated loading of condensin produces the confinement of 100–200 bp of DNA per complex (Bazett‐Jones *et al*, 2002), the (+) supercoils constrained by high concentrations of condensin might reflect incomplete loading events of stacked condensins (Fig EV5). The ATP‐dependent capacity of low condensin concentrations to restrain (−) supercoils, reported here, also contrasts with previous observations of (−) supercoils restrained by some bacterial Smcs (MukB dimers), which occurred at high protein concentrations (> 1 complex/50 bp) and in the absence of ATP (Petrushenko *et al*, 2006; Kumar *et al*, 2017).

We show that condensin activity restrains DNA (−) supercoils at different conformational stages. Prior to any round of ATP hydrolysis, nucleotide binding (AMPPNP) barely alters the DNA topology. However, following initial cycles of ATP hydrolysis, condensin restrains a ∆Lk of −0.4 and this capacity persists irrespective of further ATP consumption. Moreover, this ∆Lk restraint increases to −0.8 during each round of ATP binding and resets to −0.4 upon completion of ATP hydrolysis. Therefore, the nucleotide bound conformations of the condensin‐DNA complex are not equivalent before and after completing the initial round of ATP hydrolysis. These findings are consistent with the dual role of the ATPase activity of SMC complexes. Namely, ATP is first necessary to load the complex onto DNA (Arumugam *et al*, 2003; Murayama & Uhlmann, 2014; Wilhelm *et al*, 2015) and the subsequent cycles of ATP consumption allow the loaded complex to translocate along the DNA (Terakawa *et al*, 2017; Ganji *et al*, 2018; Davidson *et al*, 2019). The capacity to restrain negative ∆Lk values also correlates with the stability of the condensin‐DNA complex. As summarised in Fig 4I, the loaded complex (conformation captured after depleting the ATP with Apyrase) is stable, as it resists 150–300 mM salt while constraining ∆Lk of −0.4. When the ATPase heads are engaged via nucleotide binding (conformation captured by quenching ATP usage with AMPPNP), the condensin‐DNA complex is also stable while restraining ∆Lk of −0.8. Therefore, the overall restraint of −0.4 ∆Lk units and the reduced stability observed during regular ATP usage imply that condensin‐DNA interactions and restraining of (−) supercoils are weakened during intermediate stages that undergo ATP hydrolysis.

Previous studies had denoted the capacity of SMC complexes to interact with ssDNA (Hirano & Hirano, 1998; Sakai *et al*, 2003). In particular, the hinge domain has more affinity for ssDNA than dsDNA (Griese *et al*, 2010; Uchiyama *et al*, 2015); and cohesin is able to interact simultaneously with ds‐ and ss‐DNA molecules (Murayama *et al*, 2018). These observations pointed to the possibility that condensin restrained negative ∆Lk values by unwinding the DNA (∆Tw < 0). However, our results indicate that condensin does not expose an unwound region of DNA during its ATP cycle (Fig 3). Then, it seems unlikely that condensin activity is untwisting nearly a half helical turn (∆Lk −0.4) or near a full helical turn (∆Lk −0.8) of DNA, unless such unwound region of DNA is protected from the nuclease attack. The alternative way for condensin to constrain negative ∆Lk values is by stabilising a left‐handed bend or loop of DNA (∆Wr < 0). This prospect is more likely than DNA untwisting because SMC complexes necessarily bend the DNA template, in order to start and/or perform the extrusion process. In this respect, recent single‐molecule imaging has shown the preferential binding of condensin to the apex of DNA plectonemes (Kim *et al*, 2022), which might reflect its affinity for bent DNA. Other single‐molecule studies also indicated that condensin (Eeftens *et al*, 2017) and *Smc5/6* (Gutierrez‐Escribano *et al*, 2020) are able to embrace DNA plectonemes, and a cryo EM structure revealed the interaction of MukBEF with two DNA segments with a crossing angle as the one produced by a left‐handed supercoil (Burmann *et al*, 2021). All these observations support our inference that the negative ∆Lk values constrained by condensin are consequent to left‐handed bending or looping of the DNA.

Another conclusion of our study is that condensin not only restrains DNA (−) supercoils but also impedes the compensatory (+) supercoiling to spread outside the condensin‐DNA complex (Fig 5). This inference implies that condensin delimits a DNA topological domain between two separated DNA‐binding sites and then deforms (pinches) this domain into a left‐handed loop by moving the two sites towards each other (Fig 5G). As long as these two DNA interactions preclude axial rotation of the duplex, the compensatory (+) supercoiling would remain within the formed loop. Remarkably, while this loop can be relaxed by Topo I, it is not large enough to expose a (+) DNA crossover that could be relaxed by Topo II (Fig EV3). Therefore, it is unlikely that such supercoiled domain is the extruded loop, which reaches thousands of bp in length and does not appear to be supercoiled during the visualisation of loop extrusion processes (Ganji *et al*, 2018; Davidson *et al*, 2019; Kim *et al*, 2019; Golfier *et al*, 2020). However, some microscopy images have suggested that individual condensin complexes generate several supercoils (of unknown sign) away from the condensin‐DNA complex (Bazett‐Jones *et al*, 2002; Kim *et al*, 2022). In this regard, we discarded that condensin translocation generates twin supercoiled loops (Fig 6). Hence, such external supercoils could imply that condensin‐DNA complexes are able to restrain ∆Lk values much larger than those reported here. Alternatively, these supercoils could reflect preferential loading of condensin to partially relaxed DNA molecules.

In light of our results, we believe that the short DNA domain that condensin pinches into a left‐handed loop is the step segment that condensin must capture to translocate along the DNA. In this respect, former analyses of DNA extrusion step sizes of yeast condensin showed values of around 200 nm (Eeftens *et al*, 2017), but more recent measurements revealed step sizes between 17 and 40 nm (Ryu *et al*, 2022). These step sizes would correspond to DNA domains of 50–120 bp, which could be relaxed by Topo I, but not Topo II (Fig EV3).

**Figure 8:**
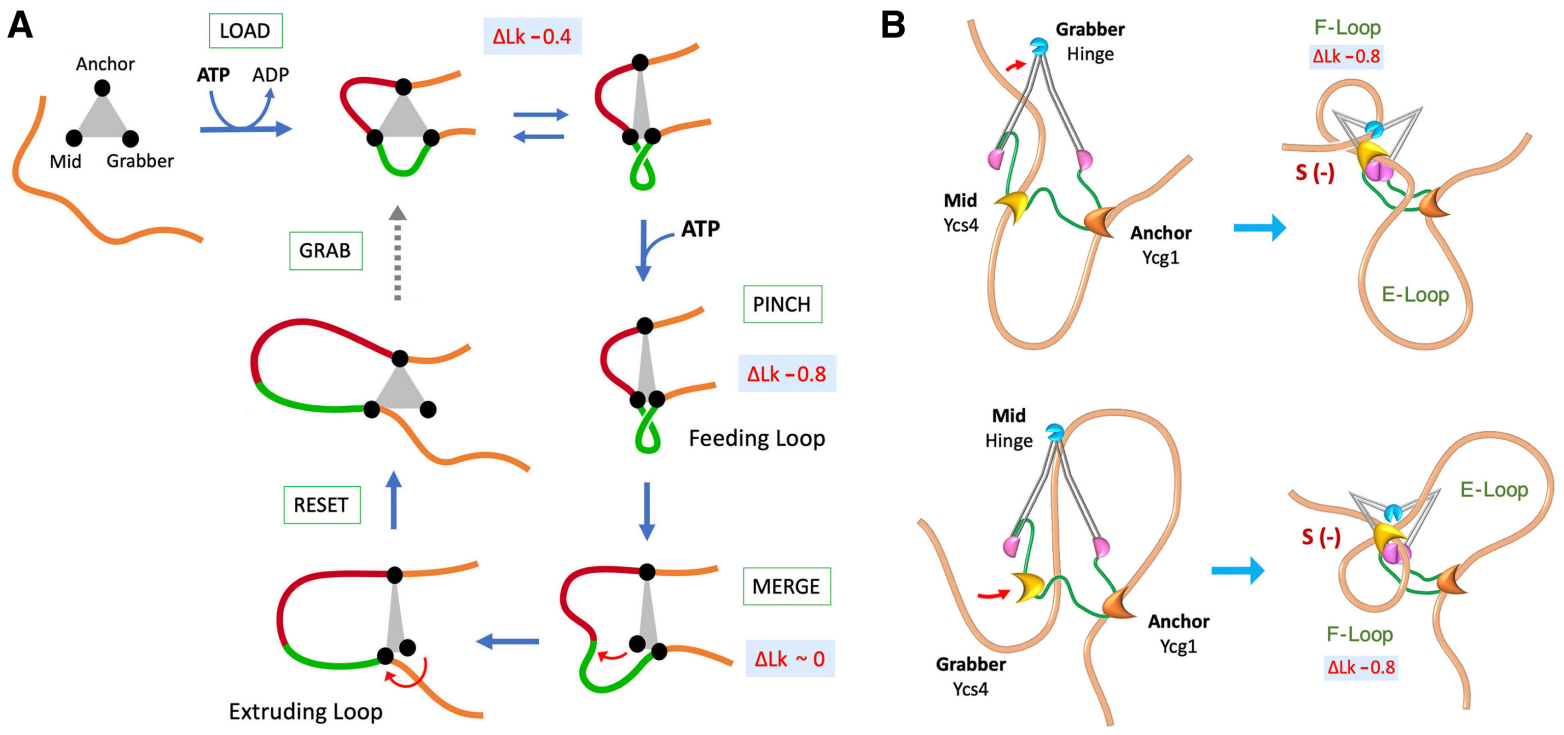
Integration of DNA topology into mechanistic models of condensin activity “Pinch and merge” mechanism of DNA loop extrusion involving three DNA‐binding modules (Anchor, Mid, Grabber). Following ATP‐mediated loading on DNA, each round of ATP usage comprises four steps (Grab, Pinch, Merge, Reset), which allow the capture of a feeding loop (green) and the subsequent enlargement of an extruded loop (red). The ∆Lk values restrained at different steps are indicated. See main text for details.Roles of the DNA‐binding modules of condensin to perform the pinch and merge mechanism. In the two models, Ycg1 provides the anchor site of the extruding loop (E‐loop), and the Ycs4 module and the hinge provide the mid and grabber sites (top) or vice‐versa (bottom). In both models, upon ATP binding, the feeding loop (F‐loop) is pinched into a (−) supercoil (∆Lk −0.8), which is delimited by the segment of DNA clamped over the engaged ATPase heads (pink).

The recurrent restraining of a short left‐handed DNA loop hints at a general mechanistic scheme of how DNA translocation steps might occur during loop extrusion (Fig 8A). This scheme involves three DNA‐binding modules: the “anchor”, the “mid” and the “grabber”. To start, initial cycles of ATP hydrolysis lead to the occupancy of these three modules. Therefore, the loaded complex delimits two distinct DNA domains, one between the anchor and the mid and another between the mid and the grabber. At this stage, oscillations of the grabber towards the mid produce a recurrent bending of the enclosed DNA. This oscillation ends upon ATP binding, which fixes the grabber close to the mid and, therefore, pinches the enclosed DNA into a tight left‐handed loop, named the “feeding loop”. Sequential hydrolysis of ATP drives two operations. First, the mid site releases its DNA. As a result, the feeding loop is no longer constrained and merges with the DNA domain formerly delimited by the mid and anchor sites. Second, the grabber transfers its DNA to the mid site. As a result, the anchor and the mid site enclose an enlarged DNA domain, which is the “extruded loop”. Reseting the complex to its loaded conformation enables the grabber to capture a new step segment of DNA and repeat the cycle.

The above mechanistic model, that we termed “pinch and merge”, explains the ∆Lk changes observed in our study. Recurrent bending of DNA in the loaded complex would restrain −0.4 units of ∆Lk, whereas the pinching step would increase this value to −0.8. The model also accounts for the distinct salt resistance of the condensin‐DNA complexes observed in our study. During the pinching step (ATP‐bound conformation) and after the completion of ATP hydrolysis (ATP exhaustion), the complex stability is high because the DNA is held by the three DNA‐binding modules. However, during the merging process (during ATP hydrolisis), the complex stability lessens because the grabber is transfering its DNA to the mid site.

The pinch and merge mechanism is also consistent with the observation that ATP binding triggers the stepping power in cohesin (Bauer *et al*, 2021) and condensin (preprint: Shaltiel *et al*, 2021; Ryu *et al*, 2022). Note that the pinching step produces the main pulling stroke on the DNA. The pinch and merge mechanism would also explain why condensin translocates faster in relaxed than in supercoiled DNA (Kim *et al*, 2022). Note that, whereas the apex of plectonemes might facilitate condensin loading, the rigidity of plectoneme stems might delay the subsequent capture and pinching of feeding loops. Another quality of the pinch and merge mechanism is that it does not involve sliding movements to tranfer the DNA from one binding site to another. This feature provides the crucial capacity of condensin to bypass nucleosomes, other condensin complexes (Kim *et al*, 2020) and obstacles much larger than the SMC's dimensions (preprint: Pradhan *et al*, 2021). Related to this, SMC complexes can extrude DNA loops without topological entrapment of DNA inside the tripartite ring (Davidson *et al*, 2019; preprint: Pradhan *et al*, 2021; preprint: Shaltiel *et al*, 2021). The pinch and merge mechanism does not require such topological entrappment. However, it is plausible that the feeding and/or extruding loops are pseudo topologically entrapped to facilitate the transfer of DNA from one module to another.

Since condensin has three main DNA‐binding modules (Appendix Fig S1), the pinch and merge mechanism can be modelled into six possible combinations depending on the role assigned to each module (Appendix Fig S12). *A priori*, since the Ycg1‐Brn1 module is peripheral and highly mobile, we expected that it would be the grabber. However, we discarded this posibility upon finding that condensin complexes lacking Ycg1 are able to restrain (−) supercoils similarly to the condensin holo‐complex (Fig 7A), even though they present little salt resitance (Fig 7B). Remarkably, these results are consistent with recent single‐molecule recordings of DNA loop extrusion that identified the Ycg1‐Brn1 module as the anchor site (Shaltiel *et al*, 2022). Then, in terms of the pinch and merge model, the hinge and the Ycs4 module would provide the grabber and mid functions (or vice‐versa; Fig 8B). If so, the DNA segment clamped between the engaged ATPase heads and Ycs4 would be one of the boundaries of the feeding loop produced by the pinching step; and the subsequent release of this DNA segment during ATP hydrolysis would initiate the merging process.

Further research, combining DNA topology with biochemical and structural analyses, might clarify how Lk changes correlate with conformational transitions of each DNA‐binding module and with the plausible topological or pseudo topological entrapment of DNA within condensin compartments. Likewise, the DNA topology approaches described here could readily indicate if analogous DNA deformations are produced by other SMC complexes. We foresee that the capacity to pinch short DNA loops is a physiological trait of most SMC complexes. However, distinct reaction settings and the stability and chirality of such loops might generate the diverse DNA supercoiling outcomes that have been puzzling the field for years.

## Materials and Methods

### Enzymes and DNA

Yeast condensin complexes were expressed and purified as reported previously (Lee *et al*, 2020). Briefly, *Saccharomyces cerevisiae* cells were transformed with a pair of 2 μ‐based high copy plasmids containing pGAL10‐YCS4 pGAL1‐YCG1 TRP1 (or pGAL10‐YCS4 to obtain ∆Ycg1 complexes) and pGAL7‐SMC4‐StrepII3 pGAL10‐SMC2 pGAL1‐BRN1‐His12‐HA3 URA3. Overexpression was induced by addition of galactose to 2%. Cell lysates were cleared by centrifugation, loaded onto a 5‐ml HisTrap™ column (GE Healthcare) and eluted with imidazole. Eluate fractions were incubated with Strep‐Tactin Superflow high‐capacity resin and eluted with desthiobiotin. Eluates were concentrated by ultrafiltration and final purification proceeded by size‐exclusion chromatography with a Superose 6 column. Purified condensin, recovered at about 3 μM concentration in 50 mM Tris–HCl pH 7.5, 200 mM NaCl, 1 mM MgCl_2_, 1 mM DTT, 5% glycerol, was snap‐frozen and stored at –80°C. Before each experiment, condensin was diluted to 300 nM concentration in 50 mM Tris–HCl pH 7.5, 1 mM EDTA, 200 mM NaCl, 1 mM DTT, 500 μg/ml BSA, 50% glycerol and kept at −20°C until mixing with DNA.

Topoisomerase I of vaccinia virus (Topo I) was expressed and purified from *E. coli* cells harbouring the expression clone pET11vvtop1 (Shuman *et al*, 1988). We defined 1 unit of Topo I as the amount of enzyme that catalysed the relaxation of 100 ng of negatively supercoiled pBR322 DNA in 5 min at 30°C in a reaction volume of 20 μl. Topoisomerase II of *S. cerevisiae* (Topo II) was expressed and purified from yeast cells carrying the expression clone YEpTOP2GAL1 (Worland & Wang, 1989). We defined 1 unit of Topo II as the amount of enzyme that catalysed the relaxation of 100 ng of negatively supercoiled pBR322 DNA in 5 min at 30°C in a reaction volume of 20 μl. Additional enzymes were from commercial sources: *E. coli* Topoisomerase I (TopA; NEB #M0301S); Nuclease P1 (NEB #M0660S); Alkaline Phosphatase (NEB #M0290); Apyrase (NEB #M0398S); Endonuclease BspQI (NEB #R06445); T4 DNA ligase (NEB #M0202T). To produce a stock of relaxed DNA, 10 μg of negatively supercoiled pBR322 (4.3 Kbp) were pre‐incubated at 30°C for 5 min in 100 μl of 25 mM Tris–HCl pH 7.5, 25 mM NaCl, 5 mM MgCl2, 1 mM DTT. Ten units of Topo I were added and the incubations proceeded for 30 min. To prepare nicked DNA, 10 μg of negatively supercoiled pBR322 were incubated with 10 units of BspQI at 55°C for 10 min in 100 μl of 25 mM Tris–HCl pH 7.5, 25 mM NaCl, 5 mM MgCl_2_, 1 mM DTT. Relaxation and nicking reactions were terminated with one volume of 20 mM EDTA and 1% SDS and extracted twice by phenol‐chloroform. DNA was recovered by EtOH precipitation and resuspended in 10 mM Tris–HCl pH 7.5, 1 mM EDTA.

### Reactions of DNA with condensin

Incubations of DNA with condensin were typically done in 20 μl of reaction buffer containing 25 mM Tris–HCl pH 7.5, 1 mM DTT, 25 mM NaCl and 5 mM MgCl_2_, unless some components were modified as indicated in specific experiments. The DNA (relaxed, nicked or negatively supercoiled pBR322) was first added at the specified final concentrations (0.3, 1 or 3 nM) followed by condensin at the specified final concentrations (0.3–240 nM). Upon preincubation at 30°C for 5 min, the DNA‐condensin mixtures were supplemented with either 1 mM ATP, 2 mM AMPPNP, 1 mM ATP subsequently quenched by 2 mM AMPPNP or 1 mM ATP subsequently exhausted by 1 unit of Apyrase. Reaction mixtures were also supplemented with either 1 unit of Topo I, 1 unit of Topo II, 1 unit of TopA or 1 unit of T4 Ligase when indicated. Reactions proceeded at 30°C for specified time periods until terminated by adding 10 μl of 20 mM EDTA, 1% SDS, 30% Glycerol, 0.3 μl of proteinase‐K (10 mg/ml) and incubated for 30 min at 50°C. Resulting 30 μl volumes were cooled at room temperature and 15 μl loaded in agarose gels for electrophoresis.

### DNA competition assays

DNA competition assays were done in a 20 μl of reaction buffer by first mixing relaxed pBR322 (0.3 nM), condensin (3 nM) and Topo I. Following 5 min incubation at 30°C, ss‐ and ds‐oligonucleotides (60 base‐pairs) were added at high concentration (100 and 500 nM) and incubations continued for 10 min at 30°C. Reactions were then supplemented with ATP (1 mM) and further incubated for 10 min. Parallel experiments were conducted by first incubating the condensin‐DNA mixtures with ATP (1 mM) for 10 min and afterwards adding the oligonucleotides and continuing the incubation for 10 min. Reactions were terminated and processed as described above.

### Immobilisation of condensin‐DNA complexes

Relaxed pBR322 (0.3 nM) condensin (3 nM) and Topo I were mixed in a 60 μl volume of buffer containing 25 mM Tris–HCl pH 7.5, 25 mM NaCl and 5 mM MgCl_2_, 0.01% Tween‐20, 10 mM Imidazole. Following a preincubation for 5 min, the mixtures were supplemented with nucleotides and incubations proceeded at 30°C for 10 min. Reaction volumes were divided into thirds of 20 μl, to which NaCl was added to reach concentrations of 50, 150 or 300 mM. Following 5 min incubation, 1 μl of His‐Tag magnetic beads (Dynabeads™ Invitrogen #10103D) was added to each tube. After 5 min incubation, reaction tubes were placed on the magnet for 2 min and the supernatant containing free DNA was recovered. To release the DNA immobilised by the his‐tagged condensin, the magnetic beads were resuspended in 20 μl of 10 mM Tris–HCl pH 7.5, 1 mM EDTA, 1% SDS, 0.3 μl of proteinase‐K (10 mg/ml) and incubated for 10 min at 50°C. The beads were centrifuged and the supernatant recovered. Ten microliter of 20 mM EDTA and 30% glycerol were added to the supernatants. Fifteen microliter of the final volumes were loaded in agarose gels.

### Nuclease P1 digestions

Relaxed pBR322 (0.3 nM), condensin (3 nM), Topo I (1 unit) and Nuclease P1 (10 units) were mixed in a 20 μl volume of reaction buffer. In the reactions that started with negatively supercoiled pBR322, Topo I was omitted. Following a preincubation for 5 min, the reaction mixtures were supplemented with nucleotides. Incubations proceeded at 30°C for indicated time periods until terminated and processed as described above.

### Electrophoresis of DNA topoisomers and calculation of ΔLk

Topoisomers of pBR322 were electrophoresed in 0.7% (w/v) agarose gels. One‐dimensional electrophoreses were carried out at 2.5 V/cm for about 20 h in TBE buffer (89 mM Tris‐borate, 2 mM EDTA) containing 0.4 μg/ml chloroquine (or as specified in figure legends). In these conditions, topoisomers around Lk° move ahead of the nicked DNA circles, and topoisomers with Lk values higher than Lk° move faster than Lk°. Two‐dimensional electrophoreses were in TBE containing 0.1 or 0.4 μg/ml chloroquine in the first dimension (2.5 V/cm for 18 h, gel top to bottom) and in TBE containing 1 μg/ml chloroquine in the second dimension (5 V/cm for 4 h, gel left to right). In these conditions, topoisomers distribute in an arch, in which Lk values increase clockwise and decrease anti‐clockwise. Gels were blot‐transferred to a nylon membrane (Amersham Hybond‐N+ #RPN203B) and probed with pBR322 DNA sequences labelled with AlkPhos Direct (GE Healthcare^®^ #GERPN3680). Chemiluminescent signals of increasing exposition periods were recorded with a cooled CCD camera (KODAK Gel Logic 1500 Imaging System) or on X‐ray films. Lk changes were analysed as described (Segura *et al*, 2018). Briefly, the midpoint of each Lk distribution, which does not necessarily coincide with the position of one DNA topoisomer, was determined by quantifying with the ImageJ software the relative intensity of non‐saturated signals of the individual Lk topoisomers. *∆Lk* was calculated as the distance (Lk units) between the midpoints of the input relaxed DNA distribution (Lk°) and of the Lk distribution restrained by condensin activity (Lk^C^). To observe the knot species produced by Topo II, the reacted DNA samples were nicked with endonuclease BspQI and examined in one‐dimensional electrophoreses as described for Lk topoisomers.

## Author contributions

**Belén Martínez‐García:** Formal analysis; investigation; methodology. **Sílvia Dyson:** Formal analysis; investigation; methodology. **Joana Segura:** Formal analysis; investigation; methodology. **Alba Ayats:** Investigation. **Pilar Gutierrez‐Escribano:** Resources. **Erin E Cutts:** Resources. **Luís Aragón:** Conceptualization; resources; funding acquisition. **Joaquim Roca:** Conceptualization; formal analysis; supervision; funding acquisition; writing – original draft; project administration; writing – review and editing.

## Disclosure and competing interests statement

The authors declare that they have no conflict of interest.

## Supporting information



Appendix

Expanded View Figures PDF

PDF+

## Data Availability

This study includes no data deposited in external repositories.
