# Supplementary material for: Condensin pinches a short negatively supercoiled DNA loop during each round of ATP usage
Source: EMBO J. 2022 Dec 19;42(3):e111913. doi: 10.15252/embj.2022111913 (PMC9890231; doi:10.15252/embj.2022111913)
Supplement: Supplementary file 1 — Appendix [file EMBJ-42-e111913-s003.pdf]

APPENDIX

Condensin pinches a short negatively supercoiled DNA loop during each round of ATP usage

Belén Martínez-García[1†], Sílvia Dyson[1†], Joana Segura[1†], Alba Ayats[1], Erin E Cutts[2], Pilar Gutierrez-Escribano[2], Luís Aragón[2], Joaquim Roca[1*]

[1] DNA Topology Lab, Molecular Biology Institute of Barcelona (IBMB), CSIC, Barcelona 08028, Spain.

[2] DNA Motors Group, MRC London Institute of Medical Sciences (LMS), London W12 0NN, UK

Contents:

Figure Number	Figure Title
Appendix Figure S1	Condensin Architecture
Appendix Figure S2	Topological method to assess DNA deformations
Appendix Figure S3	Condensin purification
Appendix Figure S4	Effect of chloroquine on the gel velocity of Lk topoisomers
Appendix Figure S5	Modelling of DNA deformations as a function of ΔWr
Appendix Figure S6	Effect of salt in the condensin capacity to restrain DNA supercoils
Appendix Figure S7	Effect of pH and temperature in condensin capacity to restrain DNA supercoils
Appendix Figure S8	Effect of ATP exhaustion on the condensin capacity to restrain DNA supercoils
Appendix Figure S9	P1 nuclease activity in relaxed and negatively supercoiled DNA
Appendix Figure S10	Relaxation of DNA with Topo I in presence of oligonucleotides
Appendix Figure S11	Condensin does not inhibit the DNA relaxation activity of Topo II
Appendix Figure S12	Plausible roles of condensin DNA binding modules to perform the "pinch and merge" mechanism

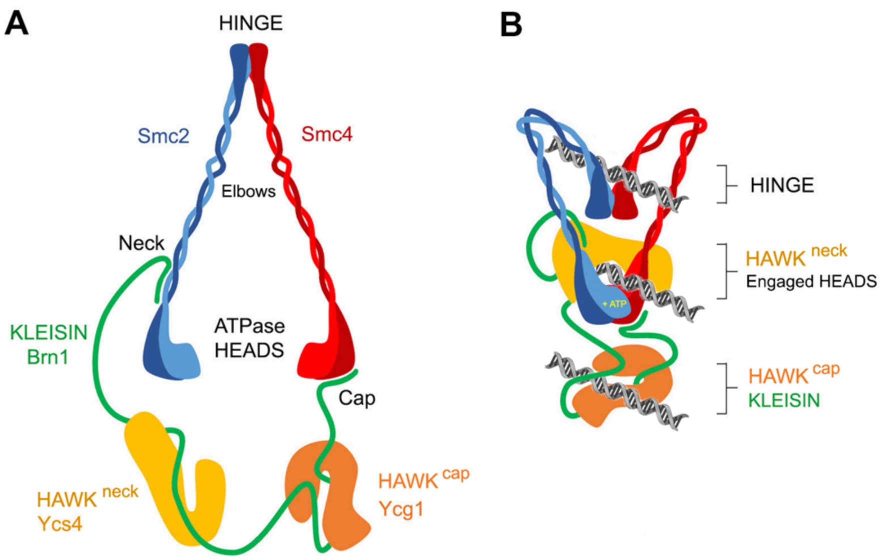

Appendix Figure S1. Condensin Architecture

(A) Structure of budding yeast condensin. The Smc subunits (Smc2, Smc4) and the kleisin (Brn1) form a protein ring. Smc2 and Smc4 each fold into a 50 nm long antiparallel coiled-coil that forms a globular “hinge” domain at its apex, whereas the amino and carboxy termini form an ATPase “head” domain at the other end. Smc2 and Smc4 dimerize via their hinge domains, while Brn1 forms a long flexible bridge connecting their head domains. The N-terminal domain of Brn1 binds to the coiled-coil "neck" region immediately adjacent to the head of Smc2, and the C-terminal domain to the head tip of Smc4 at the “cap”. The complex is completed by two HAWKs subunits. Ysc4 binds to a Brn1 region proximal to the neck ($HAWK^{neck}$), whereas Ycg1 binds to a Brn1 region proximal to the cap ($HAWK^{cap}$).

(B) DNA binding modules and conformational changes. The two ATPase heads engage with each other upon binding a pair of ATP molecules in between them. Interaction of the $HAWK^{neck}$ module with the engaged heads forms a central DNA clamping core. The $HAWK^{cap}$ module binds DNA and encircles it via a kleisin belt. A third DNA binding module is the hinge, which can reach the vicinity of the ATPase heads when the coiled coil arms bend at their elbow.

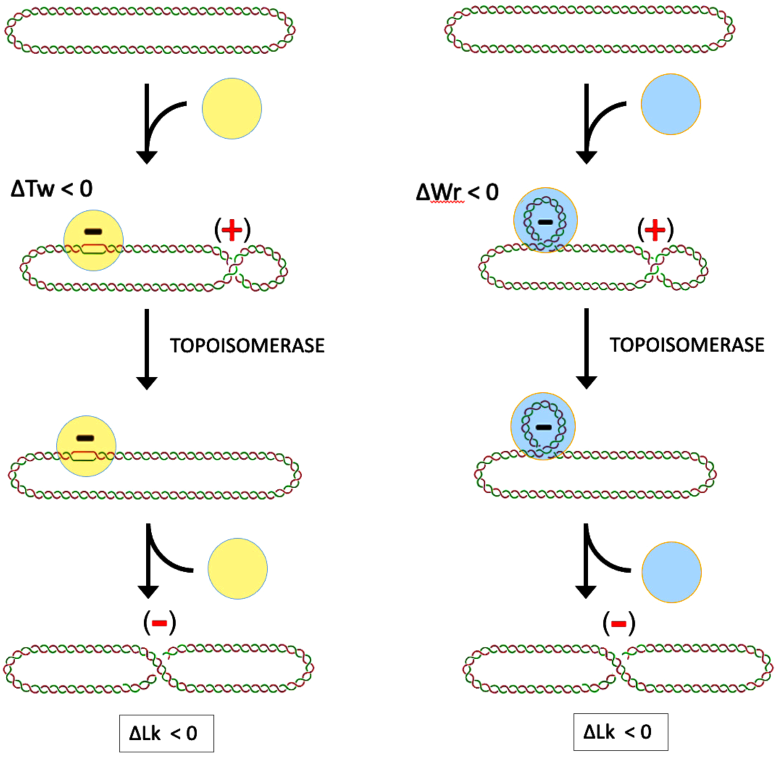

Appendix Figure S2. Topological method to assess DNA deformations

The interaction of DNA with any ligand might deform the duplex by producing ΔTw (change of helical winding) and/or ΔWr (non-planar bending). The figure illustrates, on the left, a complex that unwinds the DNA (ΔTw<0) and, on the right, a complex that bends the DNA in a left-handed manner (ΔWr<0). Since the linking number (Lk) of DNA in a closed topological domain equals the sum of the DNA's twist and writhe (Lk=Tw+Wr), the changes of Tw and/or Wr restrained by the complex will generate compensatory opposite deformations in the form of Tw and Wr. For simplicity, this is illustrated in the figure as an unconstrained (+) supercoil (ΔWr>0). Relaxation of such compensatory (+) supercoil with a topoisomerase will reset the Lk of the DNA. The resulting ΔLk value reflects the ΔTw or ΔWr deformations that were restrained by the bound complex.

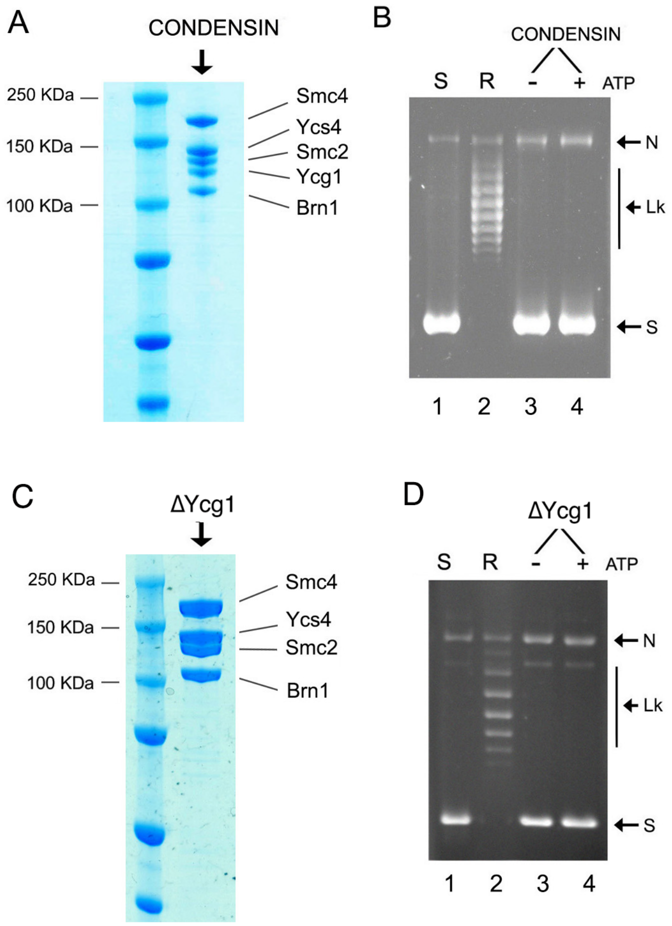

Appendix Figure S3. Condensin purification

(A) SDS-PAGE of the purified condensin pentamer (Smc2, Smc4, Brn1, Ycg1 and Ycs4).

(B) To verify that the purified condensin was free of nuclease and topoisomerase activities, 200 ng of negatively supercoiled DNA plasmid (lane 1) were relaxed with 1 unit of Topo I (lane 2), or incubated for 30 min at 30ºC with 200 ng of complex in the absence (lanes 3) or presence of 1 mM ATP (lanes 4). DNA gel electrophoresis was at 2.5 V/cm for 20 h in 0.7% agarose in TBE containing 0.2μg/mL chloroquine and stained with Ethidium. N, nicked DNA; S, negatively supercoiled DNA; Lk, distribution of relaxed topoisomers produced by Topo I.

(C) Purified condensin tetramer (ΔYcg1).

(D) Test of nuclease and topoisomerase activities conducted as in (B).

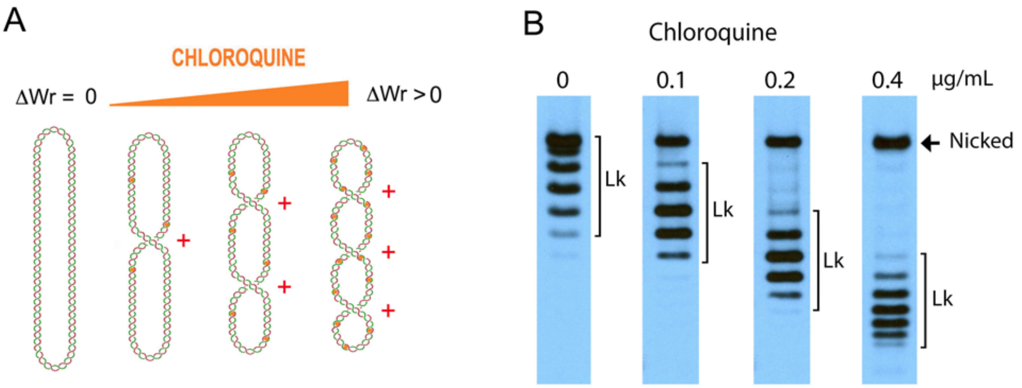

Appendix Figure S4. **Effect of chloroquine on the gel velocity of Lk topoisomers**

(A) Chloroquine intercalation unwinds the DNA double helix ($\Delta Tw<0$). In a covalently closed DNA circle, since Lk is constant, such unwinding produces positive helical tension in the chloroquine-free regions and therefore (+) supercoiling ($\Delta Wr>0$) of the DNA. Then, increasing the concentration of chloroquine increases the degree of (+) supercoiling.

(B) The gel velocity of a covalently closed DNA circle depends on its compaction volume and, therefore, on its degree of supercoiling. Accordingly, the Lk distribution of relaxed DNA will move faster when electrophoresis is conducted in the presence of chloroquine. The figure compares the electrophoretic velocity of the same distribution of Lk topoisomers that run in TBE buffer containing 0, 0.1, 0.2, and 0.4 μg/mL of chloroquine (2.5 V/cm for 18 h in 0.7% agarose).

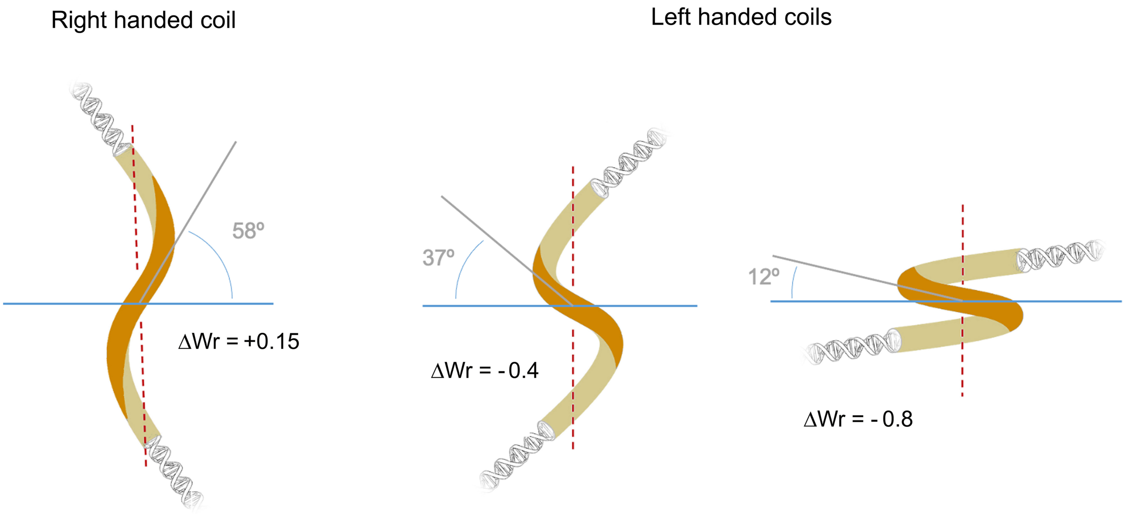

Appendix Figure S5. **Modelling of DNA deformations as a function of ΔWr**

Illustrations depict the deformation of about 120 bp of DNA to produce a ΔWr = +0.15 (right-handed coil), ΔWr = -0.4 and ΔWr = -0.8 (left-handed coils). Deformations were modelled considering that the writhe of a simple coil is given by Wr = 1 – sin ∂, were ∂ is the pitch angle of the coil (Vologodskii & Cozzarelli, 1994; Segura et al. 2018).

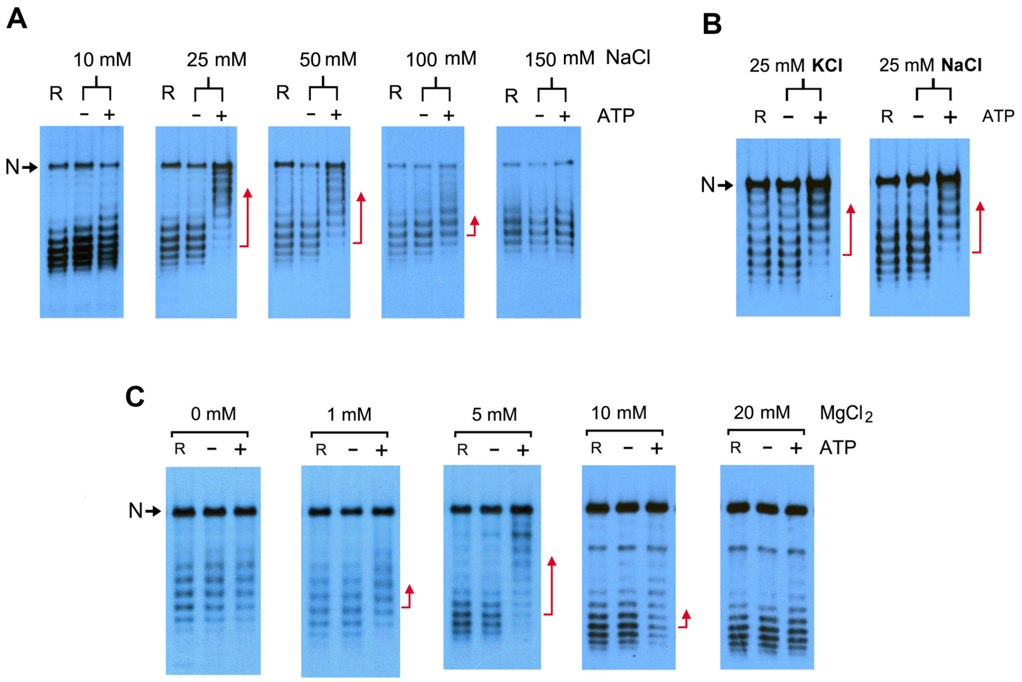

Appendix Figure S6. Effect of salt in the condensin capacity to restrain DNA supercoils

(A) Relaxed DNA (0.3 nM), condensin (3 nM) and Topo I (1 unit) were mixed in 25 mM Tris-HCl pH 7.5, 5 mM $MgCl_2$, 1 mM DTT, and different concentrations of NaCl (10, 25, 50 100, 150 mM). Mixtures were supplemented without/with ATP (1 mM) and incubated at 30ºC for 30 min.

(B) Experiment conducted as in A but containing either 25 mM NaCl or 25 mM KCl.

(C) Experiment conducted as in A but containing different concentrations of $MgCl_2$ (0, 1, 5, 10, 20 mM).

DNA electrophoreses in A, B and C were at 2.5 V/cm for 20 h in 0.7% agarose and TBE buffer containing 0.4 μg/ml chloroquine. Lanes R, DNA relaxed by Topo I (no condensin added). N, nicked circles. Red arrows denote most significant changes of ΔLk restrained by condensin in presence of ATP.

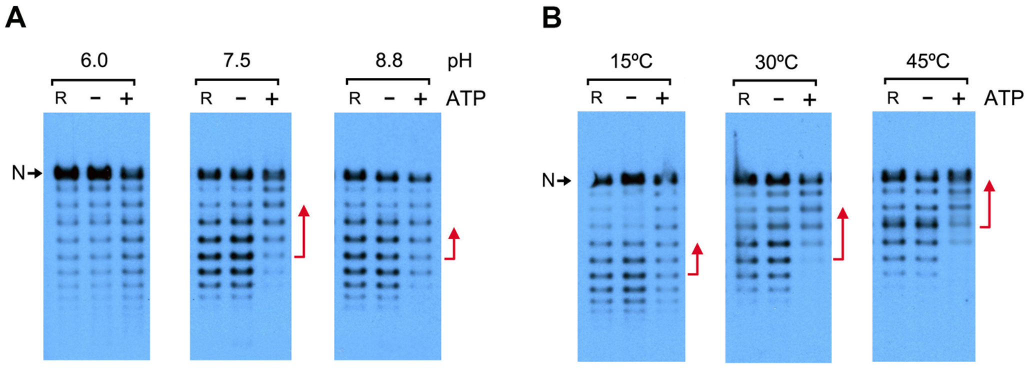

Appendix Figure S7. Effect of pH and temperature in condensin capacity to restrain DNA supercoils

(A) Relaxed DNA (0.3 nM), condensin (3 nM) and Topo I (1 unit) were mixed in 25 mM Tris-HCl, 25 mM NaCl, 5 mM $MgCl_2$, 1 mM DTT, and pH adjusted to 6.0, 7.5 or 8.8. Incubations supplemented without or with ATP (1 mM) proceeded at 30ºC for 30 min.

(B) Relaxed DNA (0.3 nM), condensin (3 nM) and Topo I (1 unit) were mixed in 25 mM Tris-HCl pH 7.5, 25 mM NaCl, 5 mM $MgCl_2$, 1 mM DTT. Incubations supplemented without or with ATP (1 mM) proceeded for 30 min either at 15ºC, 30ºC or 45ºC.

DNA electrophoreses were at 2.5 V/cm for 20 h in 0.7% agarose and TBE buffer containing 0.2 μg/ml chloroquine.

Lanes R, DNA relaxed by Topo I (no condensin added). N, nicked circles. Red arrows denote most significant changes of ΔLk restrained by condensin in presence of ATP. Note that the reference distribution of relaxed Lk topoisomers (Lanes R) also changes by the effect of pH and temperature.

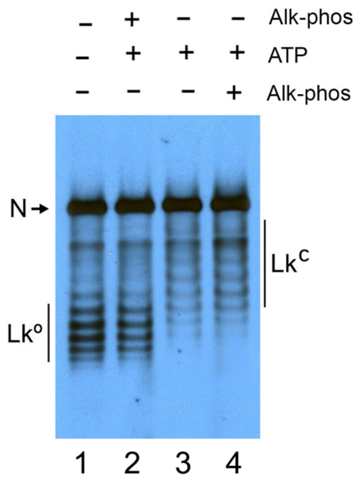

Appendix Figure S8. Effect of ATP exhaustion on the condensin capacity to restrain DNA supercoils

Relaxed DNA (0.3 nM), condensin (3 nM) and Topo I (1 unit) were mixed in 25 mM Tris-HCl pH 7.5, 25 mM NaCl, 5 mM $MgCl_2$, 1 mM DTT. Incubations proceeded at 30ºC in absence of ATP 1 mM for 10 min (lane 1), in presence of Alkaline Phosphatase and ATP 1 mM for 10 min (lane 2), ATP 1mM for 10 min (lane 3), ATP 1mM for 10 min followed by Alkaline Phosphatase for 60 min (lane 4). DNA electrophoresis was at 2.5 V/cm for 20 h in 0.7% agarose and TBE buffer containing 0.4 μg/ml chloroquine. N, nicked circles. Lk°, distribution of Lk topoisomers of relaxed DNA. Lk^{C}, resulting distribution of Lk topoisomers.

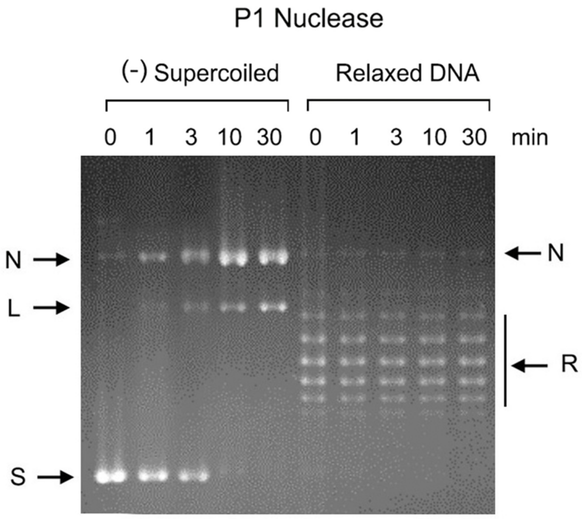

Appendix Figure S9. P1 nuclease activity in relaxed and negatively supercoiled DNA

1μg of either (-) supercoiled or relaxed DNA plasmids were incubated with 10 units of P1 nuclease in 100 μL of 25 mM Tris-HCl pH 7.5, 25 mM NaCl, 5 mM $MgCl_2$, 1 mM DTT. Volumes of 20 μL of the ongoing reactions were quenched after 0, 1, 3, 10, 30 min incubation at 30ºC. DNA electrophoresis was at 2.5 V/cm for 20 h in 0.7% agarose and TBE buffer containing 0.2 μg/ml chloroquine. The gel was stained with Ethidium. S, (-) supercoiled DNA. R, relaxed DNA. N, nicked circles. L, linear DNA.

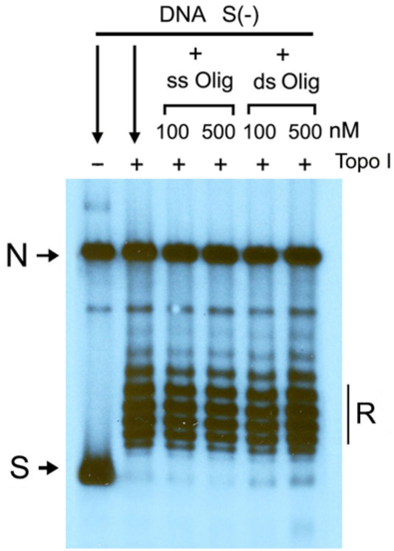

Appendix Figure S10. Relaxation of DNA with Topo I in presence of oligonucleotides

Negatively supercoiled DNA (0.3 nM) was mixed with high concentration of ss- or ds-oligos (100 and 500 nM) in 25 mM Tris-HCl pH 7.5, 25 mM NaCl, 5 mM $MgCl_2$, 1 mM DTT. Afterwards, Topo 1 (1 unit) was added to the mixtures and incubations proceeded for 10 min at 30°C. DNA electrophoresis was at 2.5 V/cm for 20 h in 0.7% agarose and TBE buffer containing 0.4 μg/ml chloroquine. S, (-) supercoiled DNA. N, nicked circles. R, relaxed DNA.

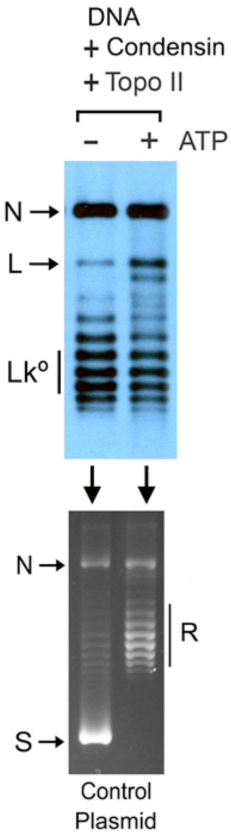

Appendix Figure S11. Condensin does not inhibit the DNA relaxation activity of Topo II.

Relaxed DNA (0.3 nM) and condensin (3 nM) were mixed in 25 mM Tris-HCl pH 7.5, 25 mM NaCl, 5 mM $MgCl_2$, 1 mM DTT, in the presence of Topo II and ATP (1 mM). Following incubation at 30ºC for 10 min, a control (-) supercoiled DNA plasmid (200 ng) was added to the mixtures and incubations continued for 10 min. DNA electrophoresis was at 2.5 V/cm for 20 h in 0.7% agarose and TBE buffer containing 0.4 μg/ml chloroquine. The top gel shows the input relaxed DNA and the bottom gel (stained with Ethidium) shows the control plasmid. Supercoiled (S), relaxed (R), nicked (N) DNA.

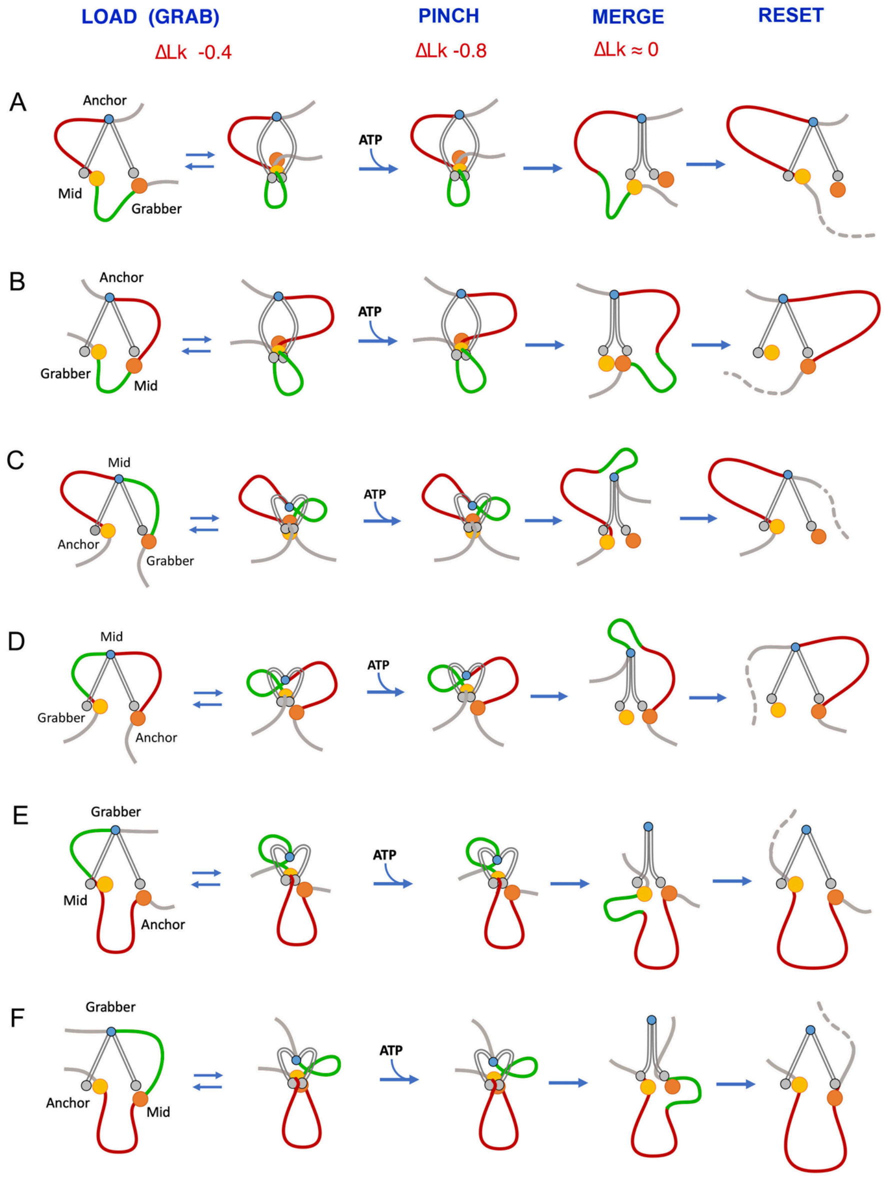

Appendix Figure S12. Plausible roles of condensin DNA binding modules to perform the "pinch and merge" mechanism

The anchor, mid, and grabber functions are assigned to the three DNA binding modules of condensin in six posible combinations (A-F). For simplicity, the kleisin subunit is not depicted and the HAWKs are represented as spheres (HAWK^{cap} in orange and HAWK^{neck} in yellow). The hinge (in blue) is acting as the anchor in A and B, as the mid site in C and D, and as the grabber in E and F. In all cases, the starting configuration is a loaded complex, in which DNA interacts with the three DNA binding modules. ATP binding produces the engagement of the Smc heads (grey) and the concomitant pinching of a feeding loop (green). ATP hydrolysis triggers the merging process and the reseting of the complex with an enlarged extruded loop (red). Dashed segments denote grabbing sites to start a new round of loop extrusion. The ΔLk restrained at different stages is indicated.
