# Supplementary material for: Condensin pinches a short negatively supercoiled DNA loop during each round of ATP usage
Source: EMBO J. 2022 Dec 19;42(3):e111913. doi: 10.15252/embj.2022111913 (PMC9890231; doi:10.15252/embj.2022111913)
Supplement: Supplementary file 2 — Expanded View Figures PDF [file EMBJ-42-e111913-s002.pdf]

# Expanded View Figures

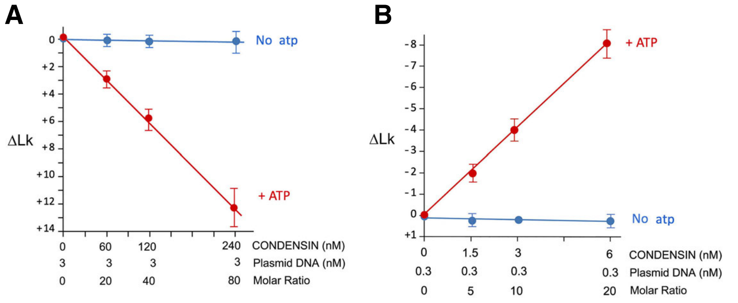


**Figure EV1. ΔLk values restrained at high and low condensin concentrations (relative to Fig 1).**

A Plot of positive ΔLk values restrained by high concentrations and molar ratios of DNA and condensin with and without ATP (mean ± SD, three technical replicates conducted as in Fig 1A).

B Plot of negative ΔLk values restrained by low concentrations and molar ratios of DNA and condensin with and without ATP (mean ± SD from three independent experiments conducted as in Fig 1C).

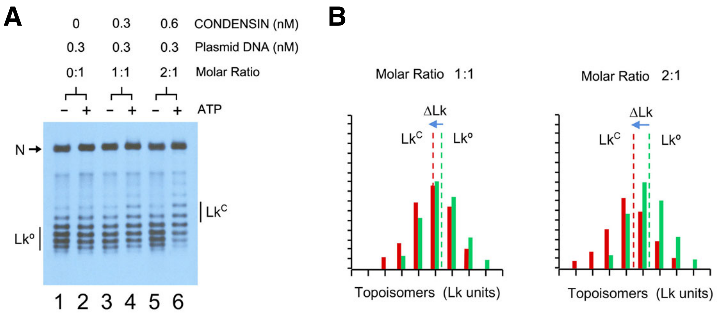


**Figure EV2. Restraining of DNA supercoils with low molar ratios of condensin (relative to Fig 1).**

A Relaxed DNA (0.3 nM), condensin (0, 0.3, 0.6 nM) and Topo I (1 unit) were mixed in 25 mM Tris–HCl pH 7.5, 25 mM NaCl, 5 mM $MgCl_2$, 1 mM DTT, with/without ATP (1 mM). Incubations proceeded at 30°C for 30 min. DNA electrophoresis was at 2.5 V/cm for 20 h in 0.7% agarose and TBE buffer containing 0.4 μg/ml chloroquine. N, nicked circles. Lk°, input distribution of Lk topoisomers of relaxed DNA. $Lk^C$, resulting distribution of Lk topoisomers.

B The histograms compare the relative intensity of individual topoisomers of the Lk distributions in lanes 3 and 4 (condensin:DNA molar ratio 1:1); and lanes 5 and 6 (molar ratio 2:1). Lk° and $Lk^C$ denote the midpoint of each Lk distribution and ΔLk the difference between them.

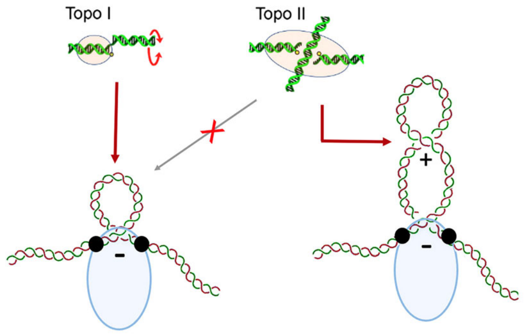


**Figure EV3. Capacity of Topo I and Topo II to relax short DNA domains (relative to Fig 5).**

The strand-rotation mechanism of Topo I requires interacting only with a short segment of DNA (< 30 bp) to relax DNA helical tension (Champoux, 2001). Conversely, DNA relaxation by the cross-inversion mechanism of Topo II requires a DNA domain long enough to facilitate the juxtaposition of two intramolecular DNA segments. Then, in the case of the DNA loop restricted by condensin, the loop length should accommodate both the restrained (–) supercoil and the compensatory (+) supercoil to be relaxed by Topo II.

©2022 The Authors

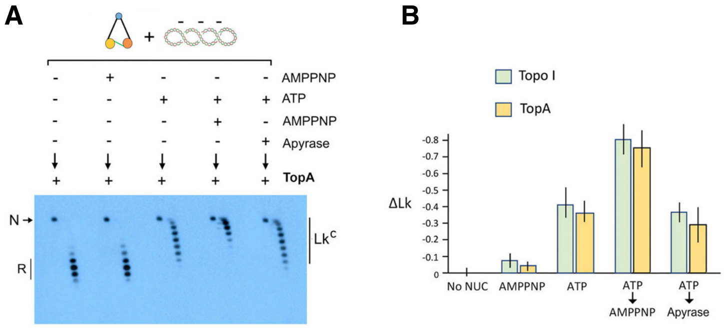


**Figure EV4. Relaxation of unconstrained (−) supercoils with TopA (relative to Fig 6).**

A (−) Supercoiled DNA (0.3 nM) was mixed with condensin (3 nM) in 25 mM Tris–HCl pH 7.5, 25 mM NaCl, 5 mM $MgCl_2$, 1 mM DTT. Following incubation in the absence or presence of the indicated nucleotides (ATP 1 mM for 30 min, AMPPNP 2 mM for 30 min, ATP for 20 min followed by AMPPNP for 10 min or ATP for 20 min followed by Apyrase for 10 min), TopA was added to relax unconstrained (−) supercoils. Incubations continued for additional 10 min. 2D-gel electrophoresis was done as in Fig 1D.

B Comparison of ΔLk values (mean ± SD, three technical replicates) restrained by condensin when unconstrained (−) supercoils were relaxed by Topo I or TopA.

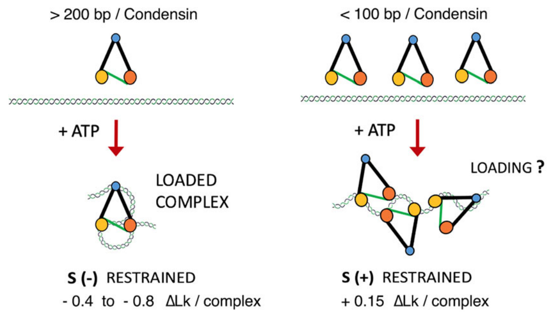


**Figure EV5. Effect of the accessible DNA length on complex loading and restraining of supercoils (relative to Fig 1).**

ATP-mediated loading of condensin leads to the confinement of 100–200 bp of DNA per complex (Bazett-Jones *et al*, 2002). Accordingly, low molar ratios of condensin to DNA (> 200 bp/complex) would allow complete loading events, which produce the restraining of (−) supercoils. High molar ratios of condensin (< 100 bp/complex) would impair proper loading and produce other DNA deformations that restrain (+) supercoils.

 ©2022 The Authors
