# Supplementary material for: Condensin pinches a short negatively supercoiled DNA loop during each round of ATP usage
Source: EMBO J. 2022 Dec 19;42(3):e111913. doi: 10.15252/embj.2022111913 (PMC9890231; doi:10.15252/embj.2022111913)
Supplement: Supplementary file 4 [file EMBJ-42-e111913-s001.pdf]

THE EMBO JOURNAL

Condensin pinches a short negatively supercoiled DNA loop during each round of ATP usage

Belén Martínez-García, Sílvia Dyson, Joana Segura, Alba Ayats, Pilar Gutierrez-Escribano, Erin Cutts, Luis Aragon, and Joaquim Roca
DOI: 10.15252/embj.2022111913

Corresponding author(s): Joaquim Roca (joaquim.roca@ibmb.csic.es)

Review Timeline:

Submission Date:	18th Jun 22
Editorial Decision:	27th Jul 22
Revision Received:	23rd Oct 22
Accepted:	5th Dec 22

Editor: Hartmut Vodermaier

Transaction Report:

(Note: With the exception of the correction of typographical or spelling errors that could be a source of ambiguity, letters and reports are not edited. Depending on transfer agreements, referee reports obtained elsewhere may or may not be included in this compilation. Referee reports are anonymous unless the Referee chooses to sign their reports.)

Thank you for submitting your study on condensin effects on DNA supercoiling for our consideration. It has now been seen by three expert referees, whose comments are copied below. As you will see, they acknowledge the importance of the subject and the potential interest of your findings, but also note some caveats, including certain discrepancies with other reports, integration with structural models/mutational data, and uncertain physiological significance. Nevertheless, should you be able to extend the in vitro studies as suggested by the referees, and to address the key specific issues raised, we would on balance be interested in pursuing a revised manuscript further for EMBO Journal publication. Important aspects in this respect would be assaying also relevant condensin mutants and some other SMC complexes (referee 1), and attempting to reconcile the data with other published findings, including the work by Kim et al that appeared while this manuscript was under review (referees 2 and 3).

Referee #1:

This elegant work by the Roca laboratory investigates changes in (plasmid) DNA topology during condensin loading and during subsequent ATP hydrolysis cycles. The study relies on a recombinant preparation of yeast condensin which has previously been shown to extrude DNA loops in vitro and on careful and extensive agarose (2D) gel analysis. Two main conclusions are drawn from the work: (1) condensin loading results in a permanent constraining of -0.4 Lk equivalents per condensin complex (in initially relaxed or supercoiled DNA). (2) during the ATP hydrolysis cycle an additional -0.4 Lk equivalents are temporarily constrained per condensin complex upon ATP-head engagement. Based on insensitivity to P1 nuclease the authors suggest that the negative supercoils are not constraint by local unwinding of DNA (in the form of ssDNA stretches). Other relevant observations are that ATP hydrolysis is needed for stable DNA binding (loading), while continuous ATP binding is not needed for maintaining DNA constraints (and DNA binding). These are novel and complementary insights that will be helpful when interpreting structural and biochemical data. An interesting side observation is that only slightly elevated protein concentrations lead to alternative outcomes (Figure 1C and F).

This is a very interesting, orthogonal, and beautifully executed investigation of condensin activity. Following points should be considered prior to publication.

Points to be considered
Condensin mutants should be investigated using the assay, in particular the safety-belt mutant will be informative. It is thought to be able to extrude loops but does not stably hold onto the DNA loading site. It would be interesting to know which steps (if any) are affected, thus revealing which DNA topology changes are absolutely needed for loop extrusion. Other mutants could be investigated as control (for example to confirm that ATP hydrolysis is needed for DNA loading).

A limitation of the manuscript in its current form is the lack of integration of the findings with known structural data and proposed models for DNA loop extrusion. This on the one hand clearly illustrates how far the field is from a comprehensive understanding of condensin-DNA interactions but also it makes it harder to draw conclusions and to make predictions. Drawing (more) realistic structural models based on prevalent hypothesis and the new findings would be helpful for the field. DNA clamping for example

should be considered to explain the -0.8 Lk change.

While the physiological relevance is hard to assess directly (in cells), the dependence of the activity on ATP hydrolysis strongly suggests that it represent a physiological reaction. Nevertheless, a strong addition to the paper would be an equivalent (but much less extensive) analysis using other SMC complexes. If the -0.4 Lk equivalent changes are conserved across SMC types it would strongly suggest that it reflects a fundamental SMC property.

Does competition with ds oligonucleotides hinder the second Lk step (observed with AMP-PNP) (which likely requires DNA clamping at ATP-engaged SMC heads)?

A curious observation is that removal of ATP by apyrase/alk-phos (after loading) appears to increase the salt-stability of DNA binding (Figure 3G). Could the authors provide an explanation?
Does it imply that ATP binding destabilizes DNA association (possibly by DNA gate opening)?

Another surprising observation is that condensin acts robustly and linearly at different protein conc. (Figure 1C and 1F) but quite abruptly changes behavior (from constraining positive supercoils to negative) at other (not so different) conditions (comparing Figure 1C to 1F). Can the authors investigate what happens at intermediary DNA and protein conc.? Is there an abrupt transition? From what to what?

Figure S12: It would be helpful to indicate where the Lk equivalents are constraint in the different models (similar to Figure 7A).

Have the authors considered using DNA ligation of nicked DNA (rather than topoisomerases) to fix plasmid DNA topology after condensin incubation (as a complementary approach)?

Referee #2:

Condensin is an ATP-dependent protein machine that compacts chromosomes during mitosis. Its ability to compact chromosomes likely stems from its activity to extrude DNA loops. Earlier studies have shown that condensin introduces positive supercoils into DNA. In the current study, Martinez-Garcia et al. provide biochemical evidence to suggest that, at low molar condensin:DNA ratios, condensin generates a negatively supercoiled DNA loop. The study relies solely on electrophoresis of DNA incubated with condensin and topoisomerases and other enzymes that nick DNA. The data appears to be consistent with their conclusions. On the other hand, the approach is indirect and the interpretation of the results could be complicated by unforeseen factors.

My concerns mainly have to do with the apparent contradictions with other published studies.

(1) The authors attempted to provide some explanation for condensin-induced positive supercoiling of DNA. The underlying mechanism for the different behaviors of condensin at low and high concentrations remains unclear to me. This is a major concern. I feel a better effort needs to be made to explain this phenomenon.

(2) A recent single-molecule study by the Dekker group shows that condensin can bind and stabilize positive supercoils and introduces supercoils (although that study can't assign a sign) away from the condensin-DNA interface. Why can't Topo II alter the link number of the condensin-DNA complex (Figure 4C; this study) if that were true? Can the authors reconcile the binding preference of condensin towards positively supercoiled DNA and the ability to pinch negatively supercoiled loops?

(3) AMPPNP could stabilize the clamped state of condensin that requires ATP binding and would therefore be expected to stabilize the condensin-DNA complex at 150 mM salt. Apyrase is expected to disrupt ATP binding by condensin and hence the clamped state of condensin. Why would the condensin-DNA complex be stable at 150 mM salt in the presence of apyrase?

(4) Their results provide some support for the model in Figure 7A, but do not provide any results that support the models drawn in 7B-E.

Referee #3:

Martínez-García et al. revisit an existing paradigm that condensin forms positive supercoils during condensation or loop extrusion. Using in vitro assays with yeast condensin, Topoisomerases, ATP and plasmid DNA, the authors thoroughly investigate the influence of condensin on DNA topology. They find that condensin causes small, but persistent negative supercoils. The resulting compensatory positive supercoil is constrained to the complex.

I would recommend the manuscript for publication, after the following comments are addressed.

Major comments:
The experiments using AMPPNP show an interesting result, which is briefly touched upon by the authors for Fig 2E. By using a non-hydrolyzing form of ATP, the amount of restrained supercoils is increased two-fold. This phenomenon can also be seen in Figure 5C and 6E. Figure 2F, lane 2 might also show a small effect with AMPPNP. Could the authors further elaborate on this finding which goes against the rationale of why a non-hydrolysis version of ATP was used?

The authors make strong statements about novelty and/or the extent of previous research, while omitting particular references. The manuscript mentions that previous experiments required high concentrations and molar ratios of condensin as a reason that the mechanistic significance has not been further investigated. Bazet-Jones et al. (2002) actually used a low concentration (or single molecule) of condensin and showed supercoiling. Whether the supercoiling was + or - was not established, but there is no mention of this paper anywhere in the manuscript.

Negative supercoiling is mentioned by Petrushenko et al. (2006) for MukB and again in Kumar et al. (2017) for MukB. Neither papers is mentioned in the manuscript, while they indicate negative supercoiling by condensin. Please incorporate and discuss these findings on negative supercoils.

Additionally, Kim et al. (NSMB 2022) recently studied the role of supercoils in condensin binding. This manuscript shows that condensin readily binds plectonemes, but that loop extrusion speed is limited for condensin on supercoiled DNA. How do the authors fit these data with their model?

Minor comments:
Figure 2B could be improved by including replicates. The text says that a plateau is reached after 10 minutes for each concentration of ATP. This however only appears to be the case for the highest concentration.

The rationale that compensatory supercoiling occurs within a domain, where TopI but not TopII can resolve this, could use further explanation. This is the same paragraph that mentions the 100{plus minus}50 bp of naked DNA, the conclusions of which are also hard to follow.

Re: EMBOJ-2022-111913

Condensin pinches a short negatively supercoiled DNA loop during each round of ATP usage

Point-by-point response (in blue) to referee comments

Referee #1:

This elegant work by the Roca laboratory investigates changes in (plasmid) DNA topology during condensin loading and during subsequent ATP hydrolysis cycles. The study relies on a recombinant preparation of yeast condensin which has previously been shown to extrude DNA loops in vitro and on careful and extensive agarose (2D) gel analysis. Two main conclusions are drawn from the work: (1) condensin loading results in a permanent constraining of -0.4 Lk equivalents per condensin complex (in initially relaxed or supercoiled DNA). (2) during the ATP hydrolysis cycle an additional -0.4 Lk equivalents are temporarily constrained per condensin complex upon ATP-head engagement. Based on insensitivity to P1 nuclease the authors suggest that the negative supercoils are not constraint by local unwinding of DNA (in the form of ssDNA stretches). Other relevant observations are that ATP hydrolysis is needed for stable DNA binding (loading), while continuous ATP binding is not needed for maintaining DNA constraints (and DNA binding). These are novel and complementary insights that will be helpful when interpreting structural and biochemical data. An interesting side observation is that only slightly elevated protein concentrations lead to alternative outcomes (Figure 1C and F).

This is a very interesting, orthogonal, and beautifully executed investigation of condensin activity. Following points should be considered prior to publication.

Points to be considered

1) Condensin mutants should be investigated using the assay, in particular the safety-belt mutant will be informative. It is thought to be able to extrude loops but does not stably hold onto the DNA loading site. It would be interesting to know which steps (if any) are affected, thus revealing which DNA topology changes are absolutely needed for loop extrusion. Other mutants could be investigated as control (for example to confirm that ATP hydrolysis is needed for DNA loading).

We had planned to extend our DNA topology assays to condensin mutants and other SMC complexes in future studies. However, we agree that testing a safety-belt mutant could be very revealing at this stage. To this end, the DNA Motors team in London purified a tetrameric condensin that lacks Ycg1 and shipped this complex to the DNA Topology Lab in Barcelona.

As described in a new section of the results and Figure 7, we found that “Ycg1 subunit is not required for condensin to restrain negative supercoils”. We explain the implications of this finding in the revised discussion (last paragraph), and modified the models depicted in Figure 8B and the abstract, accordingly.

2) A limitation of the manuscript in its current form is the lack of integration of the findings with known structural data and proposed models for DNA loop extrusion. This on the one hand clearly illustrates how far the field is from a comprehensive understanding of condensin-DNA interactions but also it makes it harder to draw conclusions and to make predictions. Drawing (more) realistic structural models based on prevalent hypothesis and the new findings would be helpful for the field. DNA clamping for example should be considered to explain the -0.8 Lk change.

As also suggested by Referee 3, we have redrawn the models in Figure 8B to illustrate how DNA clamping might concur with the formation (pinching) of a short left-handed loop that restrains -0.8 ΔLk. We also revised the models of Figure S12 by indicating the ΔLk values restrained at different stages.

To remark the limitations of our study, the revised discussion (last paragraph) reads "further research, combining DNA topology with biochemical and structural analyses, might clarify how Lk changes correlate with conformational transitions of each DNA-binding module and the plausible topological or pseudo topological entrapment of DNA within condensin compartments."

3) While the physiological relevance is hard to assess directly (in cells), the dependence of the activity on ATP hydrolysis strongly suggests that it represent a physiological reaction. Nevertheless, a strong addition to the paper would be an equivalent (but much less extensive) analysis using other SMC complexes. If the -0.4 Lk equivalent changes are conserved across SMC types it would strongly suggest that it reflects a fundamental SMC property.

We are interested in testing other SMC complexes in the future. However, our current collaborators only provided yeast condensin. We discussed this issue with the editor, who realized that extending our analysis to other SMCs was not feasible at this stage and within the scope of the present manuscript.

To comment on the physiological relevance of our findings, we added in the discussion (last sentences): "the DNA topology approaches described here could readily indicate if analogous DNA deformations are produced by other SMC complexes. We foresee that the capacity to pinch short DNA loops is a physiological trait of most SMC complexes. However, distinct reaction settings and the stability and chirality of such loops might generate the diverse DNA supercoiling outcomes that have been puzzling the field for years"

4) Does competition with ds oligonucleotides hinder the second Lk step (observed with AMP-PNP) (which likely requires DNA clamping at ATP-engaged SMC heads)?

Excellent question. We performed this new experiment and the result is shown in Figure 4C. The revised text reads: "We also found that the presence of competitor DNAs neither precluded the extra ΔLk restraint produced when ATP usage was quenched by AMPPNP (Figure 4C). Only high concentrations of ds-oligos (500 mM) slightly reduced the extra ΔLk restraint. Therefore, the transition from restraining ΔLk -0.4 to -0.8 was most likely due to a conformational change of the condensin-DNA complex rather than to the occupancy of an additional DNA binding site".

5) A curious observation is that removal of ATP by apyrase/alk-phos (after loading) appears to increase the salt-stability of DNA binding (Figure 3G). Could the authors provide an explanation? Does it imply that ATP binding destabilizes DNA association (possibly by DNA gate opening)?

There is an explanation. Note that the salt-resistance of the loaded complex (captured after ATP removal) (Figure 4H) is similar to that of the ATP bound conformation (captured by AMPPNP) (Figure 4G). However, salt-resistance is reduced during ATP usage (Figure 4F). This implies that ATP hydrolysis is what might destabilize DNA association. We clarified these notions in Figure 4I and in the revised discussion (second paragraph). Later in the discussion, we speculate that such destabilization might reflect the transfer of DNA segments during the merging stage, which is driven by ATP hydrolysis in our model.

6) Another surprising observation is that condensin acts robustly and linearly at different protein conc. (Figure 1C and 1F) but quite abruptly changes behavior (from constraining positive supercoils to negative) at other (not so different) conditions (comparing Figure 1C to 1F). Can the authors investigate what happens at intermediary DNA and protein conc.? Is there an abrupt transition? From what to what?

Referee 2 was also intrigued by this transition. Thus, we tested intermediate DNA and protein concentrations. These new results are shown in Figure 1E. "Apparently, such transition occurred when the DNA length available per complex was about 100 bp. Shorter lengths led to the restrain of (+) supercoils, whereas larger lengths allowed the restrain of (-) ones." Our interpretation of this transition is provided in the revised discussion (first paragraph) and Figure EV5.

7) Figure S12: It would be helpful to indicate where the Lk equivalents are constraint in the different models (similar to Figure 7A).

We improved Figure S12 indicating these Lk equivalents.

8) Have the authors considered using DNA ligation of nicked DNA (rather than topoisomerases) to fix plasmid DNA topology after condensin incubation (as a complementary approach)?

We did not considered using nick-ligation because, a priori, we thought it would be equivalent to using topoisomerases. However, after the referee's suggestion, we did the experiment and, surprisingly, we found that nick-ligation did not reveal a ΔLk restrain like Topo I (new Figures 5E and 5F).

We then realized that nick-ligation would only capture Lk changes if the nick was located in the small loop restrained by condensin. As most nicks would be outside the restrained loop, they would not be able to relax the compensatory (+) supercoiling of DNA. Therefore, this new result corroborated that compensatory (+) supercoiling of DNA does not spread outside the condensin-DNA complex (as we had already inferred from the Topo II experiments). We included and discussed these nick-ligation experiments in the same section of the Topo II results (Figure 5).

Referee #2:

Condensin is an ATP-dependent protein machine that compacts chromosomes during mitosis. Its ability to compact chromosomes likely stems from its activity to extrude DNA loops. Earlier studies have shown that condensin introduces positive supercoils into DNA. In the current study, Martinez-Garcia et al. provide biochemical evidence to suggest that, at low molar condensin:DNA ratios, condensin generates a negatively supercoiled DNA loop. The study relies solely on electrophoresis of DNA incubated with condensin and topoisomerases and other enzymes that nick DNA. The data appears to be consistent with their conclusions. On the other hand, the approach is indirect and the interpretation of the results could be complicated by unforeseen factors.

My concerns mainly have to do with the apparent contradictions with other published studies.

(1) The authors attempted to provide some explanation for condensin-induced positive supercoiling of DNA. The underlying mechanism for the different behaviors of condensin at low and high concentrations remains unclear to me. This is a major concern. I feel a better effort needs to be made to explain this phenomenon.

See our new experiments (Figure 1E) and insights (Figure EV5) in response to Referee 1 (point 6), who was also interested in this phenomenon.

(2) A recent single-molecule study by the Dekker group shows that condensin can bind and stabilize positive supercoils and introduces supercoils (although that study can't assign a sign) away from the condensin-DNA interface. Why can't Topo II alter the link number of the condensin-DNA complex (Figure 4C; this study) if that were true? Can the authors reconcile the binding preference of condensin towards positively supercoiled DNA and the ability to pinch negatively supercoiled loops?

We think that our results are consistent with most of the single-molecule observations recently reported by the Dekker group (Kim et al 2022).

Kim et al. observe preferential loading of condensin at the apex of plectonemes irrespective of their chirality. This is consistent with our inference that condensin would be more prone to load on a DNA segment that is already bent rather than to a straight DNA segment. We remarked this coherence in the discussion (third paragraph): "recent single-molecule imaging has shown the preferential binding of condensin to the apex of DNA plectonemes (Kim et al.), which might reflect its affinity for bent DNA"

Kim et al, also found that, although condensin readily binds to plectonemes, loop extrusion speed is limited on supercoiled DNA. This observation makes sense to us because condensin translocation by a -pinch and merge- mechanism needs DNA to be a bit loose (relaxed). In a supercoiled plectoneme, the geometry and rigidity of DNA crossings would impose a steric constraint that would delay condensin's performance to extrude loops. We included this reasoning in the revised discussion (seventh paragraph): "The pinch and merge mechanism would also explain why condensin translocates faster in relaxed than in supercoiled DNA (Kim et al). Note that, whereas the apex of plectonemes might facilitate condensin loading, the rigidity of plectoneme stems might delay the subsequent capture and pinching of feeding loops".

Lastly, Kim et al observe (via AFM) individual condensin complexes interacting with supercoiled DNA (of unknown sign) following ATP usage. They then conclude that condensin introduces supercoils away from the condensin-DNA interface. Similar images (via EM) were reported two decades ago by Bazzet Jones et al. As the referee points out, these observations contrasts with our conclusion that condensin activity does not induce supercoils outside the complex. To reconcile this discrepancy, we considered that condensin could be generating twin supercoiled loops and, therefore, no net Lk change. However, we discarded this possibility because TopA does not produce an unbalanced relaxation of (-) and (+) supercoils (Figure 6). To remark this conclusion, we changed the result caption as "Condensin activity does not generate twin supercoiled loops". In the revised discussion (fourth paragraph), we explain the plausible origin of the supercoils observed outside condensin-DNA complexes, as follows: "...some microscopy images have suggested that individual condensin complexes generate several supercoils (of unknown sign) away from the condensin-DNA complex (Bazett-Jones et al., 2002; Kim et al., 2022). In this respect, we discarded that condensin translocation generates twin supercoiled loops (Figure 6). Then, such external supercoils could mean that condensin-DNA complexes were able to restrain ΔLk values much larger than those reported here. Alternatively, these supercoils could reflect preferential loading of condensin to partially relaxed DNA molecules".

(3) AMPPNP could stabilize the clamped state of condensin that requires ATP binding and would therefore be expected to stabilize the condensin-DNA complex at 150 mM salt. Apyrase is expected to disrupt ATP binding by condensin and hence the clamped state of condensin. Why would the condensin-DNA complex be stable at 150 mM salt in the presence of apyrase?

Note that, following the exhaustion of ATP usage by Apyrase, the condensin-DNA complex is stable because it remains loaded. See our response to Referee 1 (point 5), who also asked about this matter.

(4) Their results provide some support for the model in Figure 7A, but do not provide any results that support the models drawn in 7B-E.

We agree that our previous models were too speculative. As also suggested by Referee 1, we have redrawn the models (new Figure 8B) in light of the new results that suggest the ΔYcg1 subunit is the anchor site. We illustrate how DNA clamping might concur with the formation (pinching) of a short left-handed loop that restrains -0.8 ΔLk.

Referee #3:

Martínez-García et al. revisit an existing paradigm that condensin forms positive supercoils during condensation or loop extrusion. Using in vitro assays with yeast condensin, Topoisomerases, ATP and plasmid DNA, the authors thoroughly investigate the influence of condensin on DNA topology. They find that condensin causes small, but persistent negative supercoils. The resulting compensatory positive supercoil is constrained to the complex.

I would recommend the manuscript for publication, after the following comments are addressed.

Major comments:

The experiments using AMPPNP show an interesting result, which is briefly touched upon by the authors for Fig 2E. By using a non-hydrolyzing form of ATP, the amount of restrained supercoils is increased two-fold. This phenomenon can also be seen in Figure 5C and 6E. Figure 2F, lane 2 might also show a small effect with AMPPNP. Could the authors further elaborate on this finding which goes against the rationale of why a non-hydrolysis version of ATP was used?

We revised the text of results section concerning Figure 2E and F to clarify this: "This large ΔLk restraint contrasted with the minimal effect of AMPPNP in the absence of ATP. Therefore, only following the initial cycles of ATP hydrolysis, nucleotide binding produces a conformation that further enhances the restraining of (-) supercoils". We also revised the discussion (second paragraph) to better explain why AMPPNP produces different ΔLk outcomes when it is added before ATP usage (not loaded complexes) and after ATP usage (loaded complexes). For instance, we say: "the nucleotide bound conformations of the condensin-DNA complex are not equivalent before and after completing the initial round of ATP hydrolysis".

The authors make strong statements about novelty and/or the extent of previous research, while omitting particular references. The manuscript mentions that previous experiments required high concentrations and molar ratios of condensin as a reason that the mechanistic significance has not been further investigated. Bazet-Jones et al. (2002) actually used a low concentration (or single molecule) of condensin and showed supercoiling. Whether the supercoiling was + or - was not established, but there is no mention of this paper anywhere in the manuscript.

Negative supercoiling is mentioned by Petrushenko et al. (2006) for MukB and again in Kumar et al. (2017) for MukB. Neither papers is mentioned in the manuscript, while they indicate negative supercoiling by condensin. Please incorporate and discuss these findings on negative supercoils.

We omitted these references in the initial submission to avoid over-extending our discussion. But, certainly, these previous research works merit citation. We included them and contrasted their significance with our findings. See discussion (first and fourth paragraphs) and also our concluding remarks (last sentence).

Additionally, Kim et al. (NSMB 2022) recently studied the role of supercoils in condensin binding. This manuscript shows that condensin readily binds plectonemes, but that loop extrusion speed is limited for condensin on supercoiled DNA. How do the authors fit these data with their model?

Most observations of Kim et al. fit well, even support, the pinch and merge model. See our response to Referee 2 (point 2).

Minor comments:

Figure 2B could be improved by including replicates. The text says that a plateau is reached after 10 minutes for each concentration of ATP. This however only appears to be the case for the highest concentration.

We extended the incubations to 90 min (Figure 2A and B) to corroborate the plateau.

The rationale that compensatory supercoiling occurs within a domain, where TopI but not TopII can resolve this, could use further explanation. This is the same paragraph that mentions the 100{plus minus}50 bp of naked DNA, the conclusions of which are also hard to follow.

We revised the discussion (fifth paragraph) and included Figure EV3 to better explain and illustrate why Topo I, but not Topo II, can perform in short DNA domains.

Thank you for submitting your final revised manuscript for our consideration. I am pleased to inform you that in light of the positive re-reviews by two of the original referees, we have now accepted it for publication in The EMBO Journal.

Referee #1:

The authors have addressed my comments appropriately during the revisions. The analysis of Ycg1-free yeast condensin and the topology test by nick-ligation are strong additions to the manuscript. Other added experiments are also valuable. Overall, this is excellent study that should be published without further delay.

Minor suggestions:
I do not understand why Ycg1's role in loop anchoring is mentioned in the abstract (without also mentioning that DNA restraining occurs without Ycg1)

'Restraining of (-) supercoils was robust within physiological ranges of pH (7.5) and temperature (15o to 45oC) (Appendix Fig S7).' Should 'physiological ranges of pH' be replaced by 'at a physiological pH'?

'required for patent restraining of (-) supercoils' What does 'patent' mean in this context?

Referee #3:

The authors have addressed my comments. This includes better explaining some points and also performing additional controls. Overall the manuscript has improved, and I can support publication.

A remaining minor point is that the manuscript includes quite a few typos or possibly linguistic errors (see e.g. 'mexhanism', and 'bounded' in stead of 'is bound'). May be good to have a native speaker run through the whole.

EMBO Press Author Checklist

Corresponding Author Name:	JOAQUIM ROCA
Journal Submitted to:	EMBO Journal
Manuscript Number:	ms111931

USEFUL LINKS FOR COMPLETING THIS FORM
The EMBO Journal - Author Guidelines
EMBO Reports - Author Guidelines
Molecular Systems Biology - Author Guidelines
EMBO Molecular Medicine - Author Guidelines

Reporting Checklist for Life Science Articles (updated January
This checklist is adapted from Materials Design Analysis Reporting (MDAR) Checklist for Authors. MDAR establishes a minimum set of requirements in transparent reporting in the life sciences (see Statement of Task: 10.31222/osf.io/9sm4x). Please follow the journal's guidelines in preparing your
Please note that a copy of this checklist will be published alongside your article.

Abridged guidelines for figures
1. Data
The data shown in figures should satisfy the following conditions:
- the data were obtained and processed according to the field's best practice and are presented to reflect the results of the experiments in an accurate and unbiased manner.
- ideally, figure panels should include only measurements that are directly comparable to each other and obtained with the same assay.
- plots include clearly labeled error bars for independent experiments and sample sizes. Unless justified, error bars should not be shown for technical
- if n<5, the individual data points from each experiment should be plotted. Any statistical test employed should be justified.
- Source Data should be included to report the data underlying figures according to the guidelines set out in the authorship guidelines on Data

2. Captions
Each figure caption should contain the following information, for each panel where they are relevant:
- a specification of the experimental system investigated (eg cell line, species name).
- the assay(s) and method(s) used to carry out the reported observations and measurements.
- an explicit mention of the biological and chemical entity(ies) that are being measured.
- an explicit mention of the biological and chemical entity(ies) that are altered/varied/perturbed in a controlled manner.
- the exact sample size (n) for each experimental group/condition, given as a number, not a range;
- a description of the sample collection allowing the reader to understand whether the samples represent technical or biological replicates (including how many animals, litters, cultures, etc.).
- a statement of how many times the experiment shown was independently replicated in the laboratory.
- definitions of statistical methods and measures:
  - common tests, such as t-test (please specify whether paired vs. unpaired), simple χ2 tests, Wilcoxon and Mann-Whitney tests, can be unambiguously identified by name only, but more complex techniques should be described in the methods section;
  - are tests one-sided or two-sided?
  - are there adjustments for multiple comparisons?
  - exact statistical test results, e.g., P values = x but not P values < x;
  - definition of 'center values' as median or average;
  - definition of error bars as s.d. or s.e.m.

Please complete ALL of the questions below.
Select "Not Applicable" only when the requested information is not relevant for your study.

Materials

Newly Created Materials	Information included in the manuscript?	In which section is the information available? (Reagents and Tools Table, Materials and Methods, Figures, Data Availability Section)
New materials and reagents need to be available; do any restrictions apply?	Not Applicable	

Antibodies	Information included in the manuscript?	In which section is the information available? (Reagents and Tools Table, Materials and Methods, Figures, Data Availability Section)
For **antibodies** provide the following information:<br>- Commercial antibodies: RRID (if possible) or supplier name, catalogue number and or/clone number<br>- Non-commercial: RRID or citation	Not Applicable	

DNA and RNA sequences	Information included in the manuscript?	In which section is the information available? (Reagents and Tools Table, Materials and Methods, Figures, Data Availability Section)
**Short novel DNA or RNA including primers, probes**: provide the sequences.	Not Applicable	

Cell materials	Information included in the manuscript?	In which section is the information available? (Reagents and Tools Table, Materials and Methods, Figures, Data Availability Section)
**Cell lines:** Provide species information, strain. Provide accession number in repository **OR** supplier name, catalog number, clone number, and/**OR** RRID.	Not Applicable	
**Primary cultures:** Provide species, strain, sex of origin, genetic modification status.	Not Applicable	
Report if the cell lines were recently **authenticated** (e.g., by STR profiling) and tested for mycoplasma contamination.	Not Applicable	

Experimental animals	Information included in the manuscript?	In which section is the information available? (Reagents and Tools Table, Materials and Methods, Figures, Data Availability Section)
**Laboratory animals or Model organisms:** Provide species, strain, sex, age, genetic modification status. Provide accession number in repository **OR** supplier name, catalog number, clone number, **OR** RRID.	Not Applicable	
**Animal observed in or captured from the field:** Provide species, sex, and age where possible**.**	Not Applicable	
Please detail **housing and husbandry conditions**.	Not Applicable	

Plants and microbes	Information included in the manuscript?	In which section is the information available? (Reagents and Tools Table, Materials and Methods, Figures, Data Availability Section)
**Plants:** provide species and strain, ecotype and cultivar where relevant, unique accession number if available, and source (including location for collected wild specimens).	Not Applicable	
**Microbes:** provide species and strain, unique accession number if available, and source.	Not Applicable	

Human research participants	Information included in the manuscript?	In which section is the information available? (Reagents and Tools Table, Materials and Methods, Figures, Data Availability Section)
If collected and within the bounds of privacy constraints report on age, sex and gender or ethnicity for all study participants.	Not Applicable	

Core facilities	Information included in the manuscript?	In which section is the information available? (Reagents and Tools Table, Materials and Methods, Figures, Data Availability Section)
If your work benefited from core facilities, was their service mentioned in the acknowledgments section?	Not Applicable	

Design

Study protocol	Information included in the manuscript?	In which section is the information available? (Reagents and Tools Table, Materials and Methods, Figures, Data Availability Section)
If study protocol has been **pre-registered, provide DOI in the manuscript**. For clinical trials, provide the trial registration number **OR** cite DOI.	Not Applicable	
Report the **clinical trial registration number** (at ClinicalTrials.gov or equivalent), where applicable.	Not Applicable	

Laboratory protocol	Information included in the manuscript?	In which section is the information available? (Reagents and Tools Table, Materials and Methods, Figures, Data Availability Section)
Provide DOI OR other citation details if **external detailed step-by-step protocols** are available.	Not Applicable	

Experimental study design and statistics	Information included in the manuscript?	In which section is the information available? (Reagents and Tools Table, Materials and Methods, Figures, Data Availability Section)
Include a statement about **sample size** estimate even if no statistical methods were used.	Not Applicable	
Were any steps taken to minimize the effects of subjective bias when allocating animals/samples to treatment (e.g. **randomization procedure**)? If yes, have they been described?	Not Applicable	
Include a statement about **blinding** even if no blinding was done.	Not Applicable	
Describe **inclusion/exclusion criteria** if samples or animals were excluded from the analysis. Were the criteria pre-established?<br><br>If sample or data points were omitted from analysis, report if this was due to attrition or intentional exclusion and provide justification.	Not Applicable	
For every figure, are **statistical tests** justified as appropriate? Do the data meet the assumptions of the tests (e.g., normal distribution)? Describe any methods used to assess it. Is there an estimate of variation within each group of data? Is the variance similar between the groups that are being statistically compared?	Not Applicable	

Sample definition and in-laboratory replication	Information included in the manuscript?	In which section is the information available? (Reagents and Tools Table, Materials and Methods, Figures, Data Availability Section)
In the figure legends: state number of times the experiment was **replicated** in laboratory.	Yes	Figures
In the figure legends: define whether data describe **technical or biological replicates**.	Yes	Figures

Ethics

Ethics	Information included in the manuscript?	In which section is the information available? (Reagents and Tools Table, Materials and Methods, Figures, Data Availability Section)
Studies involving **human participants**: State details of **authority granting ethics approval** (IRB or equivalent committee(s), provide reference number for approval.	Not Applicable	
Studies involving **human participants**: Include a statement confirming that **informed consent** was obtained from all subjects and that the experiments conformed to the principles set out in the WMA Declaration of Helsinki and the Department of Health and Human Services Belmont Report.	Not Applicable	
Studies involving **human participants:** For publication of **patient photos**, include a statement confirming that consent to publish was obtained.	Not Applicable	
Studies involving experimental **animals**: State details of **authority granting ethics approva**l (IRB or equivalent committee(s), provide reference number for approval. Include a statement of compliance with ethical regulations.	Not Applicable	
Studies involving **specimen and field samples:** State if relevant **permits** obtained, provide details of authority approving study; if none were required, explain why.	Not Applicable	

Dual Use Research of Concern (DURC)	Information included in the manuscript?	In which section is the information available? (Reagents and Tools Table, Materials and Methods, Figures, Data Availability Section)
Could your study fall under dual use research restrictions? Please check biosecurity documents and list of **select agents and toxins** (CDC): https://www.selectagents.gov/sat/list.htm	Not Applicable	
If you used a select agent, is the security level of the lab appropriate and reported in the manuscript?	Not Applicable	
If a study is subject to dual use research of concern regulations, is the name of the **authority granting approval and reference number** for the regulatory approval provided in the manuscript?	Not Applicable	

Reporting

The MDAR framework recommends adoption of discipline-specific guidelines, established and endorsed through community initiatives. Journals have their own policy about requiring specific guidelines and recommendations to complement MDAR.

Adherence to community standards	Information included in the manuscript?	In which section is the information available? (Reagents and Tools Table, Materials and Methods, Figures, Data Availability Section)
State if relevant guidelines or checklists (e.g., **ICMJE, MIBBI, ARRIVE, PRISMA**) have been followed or provided.	Not Applicable	
For **tumor marker prognostic studies,** we recommend that you follow the **REMARK** reporting guidelines (see link list at top right). See author guidelines, under 'Reporting Guidelines'. Please confirm you have followed these guidelines.	Not Applicable	
For **phase II and III randomized controlled trials**, please refer to the **CONSORT** flow diagram (see link list at top right) and submit the CONSORT checklist (see link list at top right) with your submission. See author guidelines, under 'Reporting Guidelines'. Please confirm you have submitted this list.	Not Applicable	

Data Availability

Data availability	Information included in the manuscript?	In which section is the information available? (Reagents and Tools Table, Materials and Methods, Figures, Data Availability Section)
Have **primary datasets** been deposited according to the journal's guidelines (see 'Data Deposition' section) and the respective accession numbers provided in the Data Availability Section?	Not Applicable	
Were **human clinical and genomic datasets** deposited in a public access-controlled repository in accordance to ethical obligations to the patients and to the applicable consent agreement?	Not Applicable	
Are **computational models** that are central and integral to a study available without restrictions in a machine-readable form? Were the relevant accession numbers or links provided?	Not Applicable	
If publicly available data were reused, provide the respective **data citations in the reference list**.	Not Applicable	
